# mTORC1-TFEB/TFE3 signaling is associated with bile acid diversification during hepatic metabolic adaptation

**DOI:** 10.1126/sciadv.aee1905

**Published:** 2026-09-25

**Authors:** Alex Zaufel, Wenfei Sun, Nunzia Pastore, Ulrike Taschler, Carina Wagner, Hubert Hackl, Judith Sommer, Dagmar Silbert-Wagner, Jan G. Hengstler, Brigitte Begher-Tibbe, Rudolf Stauber, Dagmar Kolb, Martin Troetzmueller, Benjamin Gottschalk, Chelsea Thorsheim, Eva-Lena Stange, Mathias W. Hornef, Olga Pivovarova-Ramich, Andreas F. H. Pfeiffer, Kylie P. Matchett, Anne Loft, Stephan Herzig, Neil C. Henderson, Andrea Ballabio, Zoltan Arany, Christian Wolfrum, Peter Fickert, Tarek Moustafa

**Affiliations:** ^1^ Division of Gastroenterology and Hepatology, Department of Internal Medicine, Medical University of Graz; Graz, Austria.; ^2^ Institute of Food, Nutrition and Health, ETH Zurich, Schwerzenbach, Switzerland.; ^3^ Division of Endocrinology, Department of Medicine, Stanford University School of Medicine, Stanford University, Palo Alto, CA, USA.; ^4^ Telethon Institute of Genetics and Medicine (TIGEM); Pozzuoli, Italy; Medical Genetics Unit, Department of Medical and Translational Science, Federico II University, Naples, Italy.; ^5^ Department of Translational Medicine, Medical Genetics, Federico II University, Naples, Italy.; ^6^ Institute of Molecular Biosciences, University of Graz, Graz, Austria.; ^7^ Institute of Bioinformatics, Biocenter, Medical University of Innsbruck, Innsbruck, Austria.; ^8^ Department of Toxicology, Leibniz Research Centre for Working Environment and Human Factors, Technical University Dortmund, Dortmund, Germany.; ^9^ Core Facility, Ultrastructure Analysis, Center for Medical Research, Medical University of Graz, Graz, Austria.; ^10^ Gottfried Schatz Research Center, Molecular Biology and Biochemistry, Medical University of Graz, Graz, Austria.; ^11^ Core Facility, Mass Spectrometry, Center for Medical Research, Medical University of Graz, Graz, Austria.; ^12^ Cardiovascular Institute Perelman School of Medicine, University of Pennsylvania, Philadelphia, PA, USA.; ^13^ Institute of Medical Microbiology, Rheinisch-Westfälische Technische Hochschule Aachen University Hospital;,Aachen, Germany.; ^14^ Department of Molecular Metabolism and Precision Nutrition, German Institute of Human Nutrition Potsdam-Rehbruecke, Nuthetal, Germany.; ^15^ Charité–Universitätsmedizin Berlin, Corporate Member of Freie Universität Berlin and Humboldt-Universität zu Berlin, Department of Endocrinology and Metabolism, Berlin, Germany.; ^16^ German Center for Diabetes Research (DZD), München-Neuherberg, Germany.; ^17^ Centre for Inflammation Research, Institute for Regeneration and Repair, Edinburgh BioQuarter, University of Edinburgh, Edinburgh, UK.; ^18^ Institute for Diabetes and Cancer, Helmholtz Center Munich, Neuherberg, Germany.; ^19^ Functional Genomics & Metabolism Research Unit, Department of Biochemistry and Molecular Biology, University of Southern Denmark, Odense, Denmark.; ^20^ MRC Human Genetics Unit, Institute of Genetics and Cancer, University of Edinburgh, Edinburgh, UK.; ^21^ Department of Molecular and Human Genetics, Baylor College of Medicine, and Jan and Dan Duncan Neurological Research Institute, Texas Children’s Hospital, Houston, TX, USA.

## Abstract

The mechanistic target of rapamycin complex 1 (mTORC1) integrates nutrient and hormonal cues to regulate hepatic lipid metabolism with major implications for metabolic dysfunction-associated steatotic liver disease (MASLD). Here, we show that altered hepatic mTORC1–TFEB/TFE3 signaling is associated with coordinated remodeling of bile acid (BA) metabolism during metabolic adaptation. Our data support a model in which cross-talk between mTORC1 and TFEB/TFE3 is associated with divergent regulation of bile acid synthesis and transformation. Depending on the mTORC1 signaling state, changes in hepatic *Cyp2c70* and *Cyp8b1* expression, together with altered cholesterol trafficking, were associated with shifts toward non–12-OH or 12-OH bile acid species. These effects were attenuated or reversed by *Tfe3* deletion or rapamycin treatment. Furthermore, protein restriction (which inhibits mTORC1) similarly reshaped the BA profile in mice and correlated with improved metabolic outcomes in MASLD patients. Together, these findings uncover BA homeostasis as an integral component of the metabolic adaptations orchestrated by mTORC1, underscoring a link between nutrient signaling and metabolic liver disease.

## INTRODUCTION

The mechanistic target of rapamycin complex 1 (mTORC1) integrates diverse environmental signals to coordinate regulation of growth and metabolism. Growth factors and nutrients converge on mTORC1 through several upstream regulators, including the Rag guanosine triphosphatases (GTPases) (RagA/B and RagC/D), Ras homolog enriched in brain (RHEB), GTPase-activating proteins (GAPs), AMP-activated protein kinase (AMPK), the Folliculin complex (FLCN-FNIP), and the tuberous sclerosis complex (TSC). In particular, the Ragulator complex (composed of Lamtor1-5) mediates the targeting of mTORC1 to the lysosomal surface and is necessary for its activation by amino acids (*1*). mTORC1 signaling can be broadly divided into two functional outputs. The “canonical” branch refers to the nutrient- and growth factor–responsive pathway classically monitored by phosphorylation of ribosomal protein S6 kinase (S6K) and eukaryotic translation initiation factor 4E-binding protein 1 (4E-BP1), which mediate protein synthesis (*2*, *3*). In contrast, the “noncanonical” branch refers to mTORC1-dependent regulation of the MiT/TFE family members transcription factor EB (TFEB) and transcription factor E3 (TFE3), master regulators of lysosomal biogenesis and autophagy (*4*, *5*). Recent studies suggest that TFEB and TFE3 are regulated by mTORC1 through mechanisms distinct from the classical S6K and 4E-BP1 axis, instead involving the FLCN-RagC/D pathway (*6*). These canonical and noncanonical branches of mTORC1 signaling act in concert with transcription factors (TFs) such as sterol regulatory element-binding protein 1 and 2 (SREBP1/2) and peroxisome proliferator–activated receptor alpha (PPARα) to regulate hepatic de novo lipogenesis (DNL) and fatty acid oxidation (FAO), thereby preparing the liver to respond efficiently to food intake and adapt metabolically during fasting. Chronic overnutrition, however, can lead to excessive hepatic lipid accumulation, with sustained DNL and mTORC1 hyperactivation—hallmarks of obesity, insulin resistance (IR), and type 2 diabetes (T2D) (*7*). Because mTORC1 senses many nutritional and hormonal inputs, additional mechanisms must help translate these signals into appropriate metabolic outputs during feeding and fasting. We hypothesized that bile acid (BA) metabolism may be one such mechanism.

BAs, synthesized from cholesterol in the liver, are intimately tied to fasting and refeeding cycles because they enable efficient intestinal lipid absorption. Since the composition of dietary nutrients and metabolic hormones influence BA synthesis, it is conceivable that hepatic metabolism is dynamically interdependent with changes in the BA pool size and composition (*8*). This is relevant because individual BA species differ in their metabolic effects. Certain BA species have been positively associated with beneficial dietary interventions and inversely correlated with IR, T2D, and obesity (*9*, *10*). By contrast, 12α-hydroxylated BA species (12-OH BAs) are found to be enriched in metabolic dysfunction–associated steatotic liver disease (MASLD) and advanced steatohepatitis (MASH) (*11*–*15*). In addition to excessive triacylglyceride (TAG) accumulation, dysregulated cholesterol and oxysterol metabolism is increasingly recognized as a key driver of MASH. The transport of cholesterol between intracellular compartments also determines the synthesis of specific BAs, whose biochemical and biophysical properties influence lipid solubilization and hepatic gene regulation (*8*, *16*). Because intracellular cholesterol trafficking affects which BAs are produced, it may provide a mechanistic link between nutrient sensing, mTORC1 activity, and BA composition. Sterol-sensing organelles, including the endolysosomal network, direct their lipid processing capacities toward mTORC1 activation (*17*), underscoring the therapeutic potential of targeting lysosomal mTORC1 signaling pathways in MASLD and MASH. Recent studies suggest that selective inhibition of mTORC1 protects against hepatic steatosis via TFEB/TFE3 activation, which enhances FAO and suppresses SREBP1-mediated DNL (*18*). The loss of mTORC1 activity leads to nuclear translocation of TFEB/TFE3 and suppresses DNL, suggesting additional mechanisms that account for differences in liver inflammation, steatosis, fibrosis, and tumorigenesis among mTORC1-related mouse models subjected to obesogenic, MASLD/MASH-inducing diets (*18*–*22*).

Here, we investigate whether altered mTORC1 signaling is associated with coordinated changes in hepatocyte identity, lipid metabolism, and BA composition. Using complementary genetic and dietary models, we distinguish BA phenotypes linked to reduced canonical mTORC1 signaling from those observed when MiT/TFE activity occurs with preserved or hyperactive canonical mTORC1 output. Our findings support a model in which mTORC1-TFEB/TFE3 cross-talk contributes to context-dependent remodeling of BA metabolism during hepatic metabolic adaptation.

## RESULTS

### Lamtor2 maintains structural and functional integrity of the liver

Liver-specific deletion of *Lamtor2* (Ltor2-LKO) resulted in severely distorted liver architecture, characterized by a periportal (PP) population of small hepatocytes with reduced cell size, pyknotic nuclei, and clear, glycogenated cytoplasm, as well as normal-appearing hepatocyte foci with preserved mTORC1 activation (Fig. 1, A and B, and fig. S1A). Ultrastructural analysis demonstrated mitochondrial swelling and cristae degradation (fig. S1B). Ltor2-LKO mice exhibited only mild liver injury, and body and liver weight were unaffected (Fig. 1, C to E). Hepatic glycogen and serum glucose levels were lower despite functional AKT signaling (Fig. 1, F and G, and fig. S1, C to F), while insulin and gluconeogenesis gene expression remained unchanged (Fig. 1, H and I). Glucose storage occurred in the PP small hepatocytes, contrasting regular storage in the pericentral (PC) areas of wild-type (WT) mice (fig. S1C). This hepatocyte population dominated the livers of young animals, but was gradually replaced by normal-appearing hepatocyte foci over time (fig. S1, G to L). In the livers of those animals, canonical mTORC1 signaling remained sensitive to rapamycin (fig. S1M). Ltor2-LKO mice developed a tumorigenic phenotype with bile duct hyperplasia in 80% (*n* = 4/5) of ∼1.5-year-old animals (fig. S1I).

**Fig. 1.**
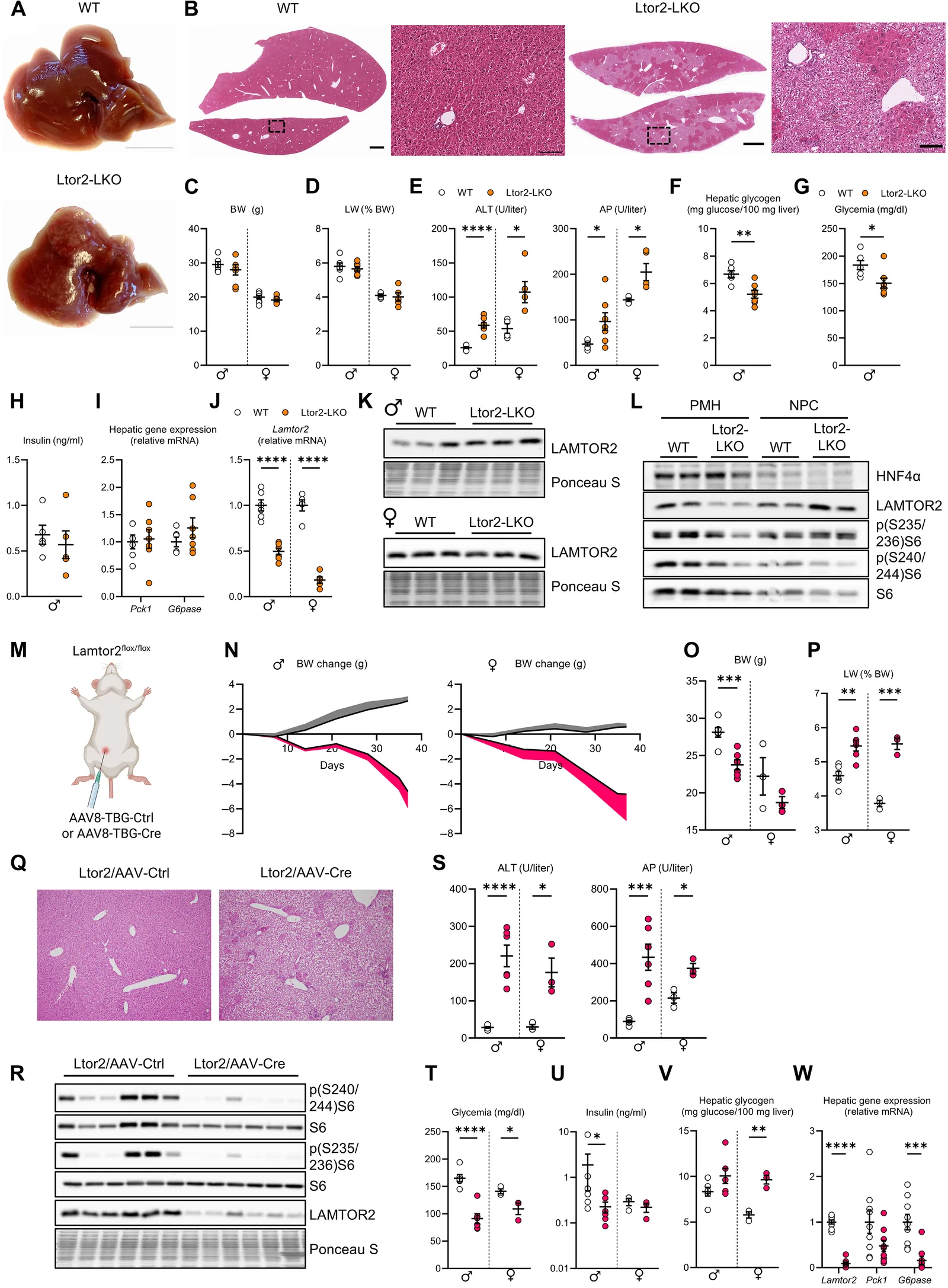
Lamtor2 maintains structural and functional integrity of the liver. (**A**) Gross liver appearance of WT and Ltor2-LKO mice. Scale bars, 10 mm. (**B**) Representative hematoxylin and eosin (H&E)–stained liver sections from WT and Ltor2-LKO mice. Scale bars, 1 mm and 100 μm in zoomed images. (**C** to **E**) Body weight, relative liver weight, and serum ALT/AP levels in 10- to 16-week-old WT and Ltor2-LKO mice fed normal chow and euthanized ad libitum (*n* = 5 to 7). (**F** to **I**) Hepatic glycogen, serum glucose, serum insulin, and hepatic *Pck1*/*G6pase* expression in male WT and Ltor2-LKO mice (*n* = 5 to 7). (**J**) Hepatic *Lamtor2* mRNA expression in WT and Ltor2-LKO mice (*n* = 5 to 7). (**K**) Hepatic LAMTOR2 protein expression in WT and Ltor2-LKO mice. (**L**) pS6, LAMTOR2, and HNF4α protein expression in primary mouse hepatocytes and nonparenchymal cells isolated from WT and Ltor2-LKO livers (*n* = 2 mice per genotype). (**M**) Experimental scheme for AAV-mediated adult hepatocyte *Lamtor2* deletion. Created in BioRender. A. Zaufel (2026); https://BioRender.com/vd8qjiy. (**N**) Body-weight change after injection of AAV8-TBG-Cre or control virus in male and female *Lamtor2*^flox/flox^ mice. (**O** and **P**) Terminal body weight and relative liver weight four weeks after virus injection (*n* = 3 to 6). (**Q**) Representative H&E-stained liver sections from Ltor2/AAV-Ctrl and Ltor2/AAV-Cre mice. Scale bars, 100 μm. (**R**) pS6 and LAMTOR2 protein expression in bulk liver extracts. (**S**) Serum ALT/AP levels four weeks after virus injection (*n* = 3 to 6). (**T** to **W**) Serum glucose, serum insulin, hepatic glycogen, and hepatic *Lamtor2*, *Pck1*, and *G6pase* expression four weeks after virus injection (*n* = 3 to 6). Data are means ± SEM. Statistical significance was calculated by two-tailed unpaired Student’s *t* test. *P < 0.05, **P < 0.01, ***P < 0.001, and ****P < 0.0001.

Bulk liver RNA expression confirmed the loss of *Lamtor2*, but protein levels remained unchanged (Fig. 1, J and K). This was due to higher LAMTOR2 expression in nonparenchymal cells (NPCs) (Fig. 1L), which express constitutively higher levels compared to hepatocytes. Unlike Ltor2-LKO mice, the deletion of *Lamtor2* in adult hepatocytes using adeno-associated virus AAV serotype 8 (AAV8) expressing Cre recombinase (Ltor2/AAV-Cre) resulted in a gradual decrease in body weight and an increase in liver weight (Fig. 1, M to P) and was accompanied by hepatocytes with a clear cytoplasmic appearance, loss of LAMTOR2 and pS6 protein, and notable liver injury in both male and female mice (Fig. 1, Q to S). Serum glucose and insulin levels were lower, along with reduced expression of glucose metabolic genes, whereas total liver glycogen significantly increased as compared with Ltor2-LKO mice (Fig. 1, T to W), indicating altered hepatocellular carbohydrate storage (fig. S1C). The clear cytoplasmic appearance was therefore interpreted cautiously and not equated with steatosis based on morphology alone. Overall, our findings suggest that the absence of a functional Ragulator complex rearranges liver architecture and impairs central metabolic functions of hepatocytes deficient in *Lamtor2*.

### Single-nuclei genomics identifies transcriptional heterogeneity across the hepatic parenchyma

We next used R26-LSL^flox/flox^-tdTomato (tdTom) reporter mice to examine hepatocellular differentiation processes and analysis of cell behavior during chronic and acute *Lamtor2* deletion. Native tdTom fluorescence was markedly weaker expressed in the PP small-hepatocyte population of Ltor2-LKO livers, defined morphologically by reduced cell size, pyknotic nuclei, and clear glycogenated cytoplasm, while these cells remained positive by anti–red fluorescent protein (RFP) staining. This indicates that reduced native tdTom signal in chronic Ltor2-LKO livers reflects loss of tdTom fluorescence rather than absence of recombination (Fig. 2A, top panels). In contrast, Ltor2/AAV hepatocytes showed uniform tdTom expression, including in morphologically small hepatocytes (Fig. 2A, bottom panels), indicating that chronic embryonic/neonatal versus acute adult loss of *Lamtor2* is associated with distinct hepatocellular phenotypes.

**Fig. 2.**
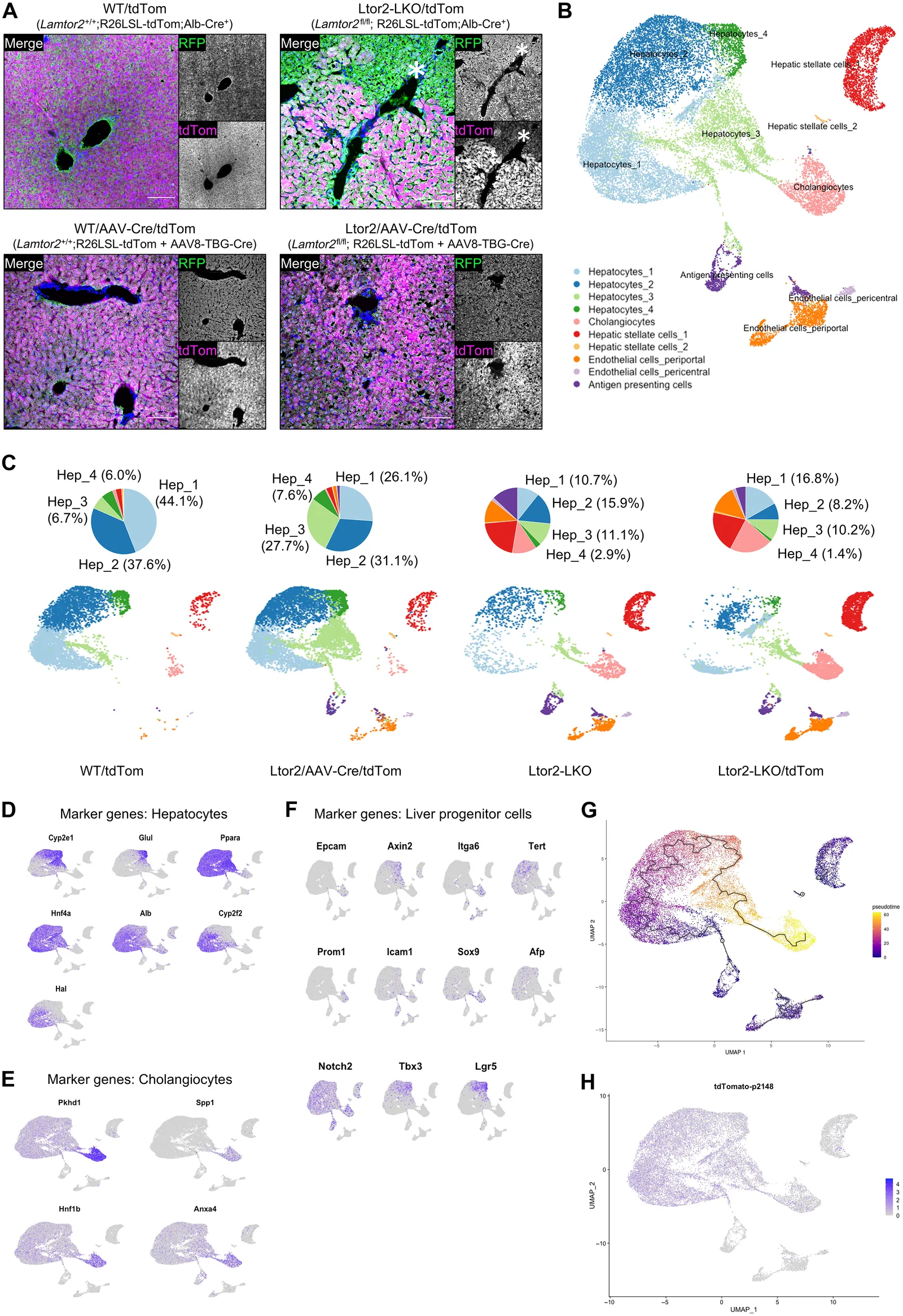
snRNA-seq analysis reveals transcriptional heterogeneity across the hepatic parenchyma. (**A**) Lineage tracing after chronic Alb-Cre-mediated or acute AAV-Cre-mediated *Lamtor2* deletion. Confocal images of liver sections from the four indicated tdTomato reporter genotypes show native tdTomato fluorescence (tdTom) and anti-tdTomato staining (RFP). In chronic Ltor2-LKO livers, native tdTom fluorescence is selectively reduced in the PP small-hepatocyte population, defined morphologically by reduced cell size, pyknotic nuclei, and clear glycogenated cytoplasm, whereas anti-RFP staining remains detectable. Asterisks indicate representative RFP-positive hepatocytes with reduced or absent native tdTom fluorescence. In contrast, tdTom signal remains uniform after acute AAV-Cre-mediated *Lamtor2* deletion. Homogeneous recombination/gene knockout was observed in >99% of hepatocytes in the tested genotypes. Images are representative of at least three mice per condition. Scale bars, 100 μm. (**B**) Uniform manifold approximation and projection (UMAP) visualization of 23,590 liver nuclei isolated from four genotypes. Unsupervised clustering identified 10 manually annotated liver cell populations. For the snRNA-seq experiment, mice were approximately 16 to 20 weeks old at tissue collection; AAV-treated mice were analyzed 4 weeks after virus injection. (**C**) Cluster composition in each genotype. Pie charts show the relative contribution of each annotated population to total nuclei. Hepatocyte subpopulations are labeled Hep_1 to Hep_4. (**D** to **F**) Feature plots showing marker genes for hepatocytes, cholangiocytes, and LPCs. (**G**) Hepatocyte differentiation/reprogramming trajectory visualized by UMAP and colored by pseudotime. (**H**) Feature plot showing tdTomato expression.

Single-nucleus RNA sequencing (snRNA-seq) analysis from livers of floxed, Ltor2-LKO, and Ltor2/AAV-Cre animals followed by unsupervised clustering identified 10 different liver cell populations, including four distinct hepatocyte subpopulations (Fig. 2B and fig. S2, A and B). Manual annotation demonstrated that Hep_2 denotes PC to midlobular hepatocytes, Hep_4 specifically the first layers of PC hepatocytes and Hep_1 PP hepatocytes. Hep_3 represents a population of hepatocytes with genes such as, *Eci2*, *Large1*, *Onecut1*, and *Mmp27*, comprising one-third of Ltor2/AAV hepatocytes (Fig. 2C and fig. S2A). In contrast to Ltor2/AAV-Cre, ∼35% of all sequenced nuclei in Ltor2-LKO livers expressed *Pkhd1*, *Spp1*, as well as *Prom1* and *Sox9*, indicating that hepatocytes in Ltor2-LKO mice have acquired biliary or liver progenitor-like cells (LPCs) gene signatures (Fig. 2, D to F, and fig. S2, C to E). Specific changes in gene expression along the pseudotime trajectory—with exclusive enrichment of tdTom RNA—showed progression along a porto-central axis from Hep_2-Hep_4-Hep_3 subpopulations (Fig. 2, G and H).

Comprehensive characterization of biological processes from the hepatocellular subpopulations (fig. S3, A to H) demonstrated that lipid biosynthesis and small GTPase-mediated signaling were specifically enriched in Hepa_3 of Ltor2/AAV (fig. S3I), BA biosynthesis and amino acid catabolism specific in Hepa_5 from Ltor2-LKO (fig. S3J), and fatty acid catabolism and oxidation enriched in both hepatocyte populations (fig. S3, I and J). Thus, our data suggest that hepatocellular plasticity and metabolic differentiation dynamics depends on a functional Ragulator/mTORC1 complex.

### Lamtor2 deficiency reprograms hepatocytes to an early postnatal-like metabolic state

The LAMTOR (“Ragulator”) complex functions upstream of TFs TFEB/TFE3, which are required for cellular differentiation (*23*). We used adenoviruses expressing either green fluorescent protein (GFP) or CMV-Cre to delete *Lamtor2* in primary mouse hepatocytes (PMHs) isolated from Ltor2/floxed mice. *Lamtor2* expression was decreased by more than 90% (Fig. 3A). The robust induction of *Rragd* mRNA pointed toward TFEB/TFE3 activation and coincided with an increase in *Raptor*, *Rictor*, and *Tfe3* expression levels (Fig. 3A). Representative confocal images of Ltor2/AAV liver sections showed nuclear TFEB, TFE3, and SRY-Box transcription factor 9 (SOX9) staining in many HNF4A-positive hepatocytes (Fig. 3B). The mRNA expression of pan-hepatocyte marker genes (*Cyp2e1*, *Alb*, and *Arg1*) was significantly reduced in Ltor2/AAV hepatocytes, alongside the mild activation of NOTCH (*Hes1*) and YAP/TAZ (*Cyr61*) signaling, potentially affecting liver cell fate (Fig. 3, C and D). Representative images further showed scattered pS6-positive cells in Ltor2/AAV-Cre livers despite preserved PP and PC marker expression. In Ltor2-LKO livers, nuclear TFEB, TFE3, and SOX9 staining was largely confined to the small-hepatocyte population (Fig. 3B and fig. S4, A to C).

**Fig. 3.**
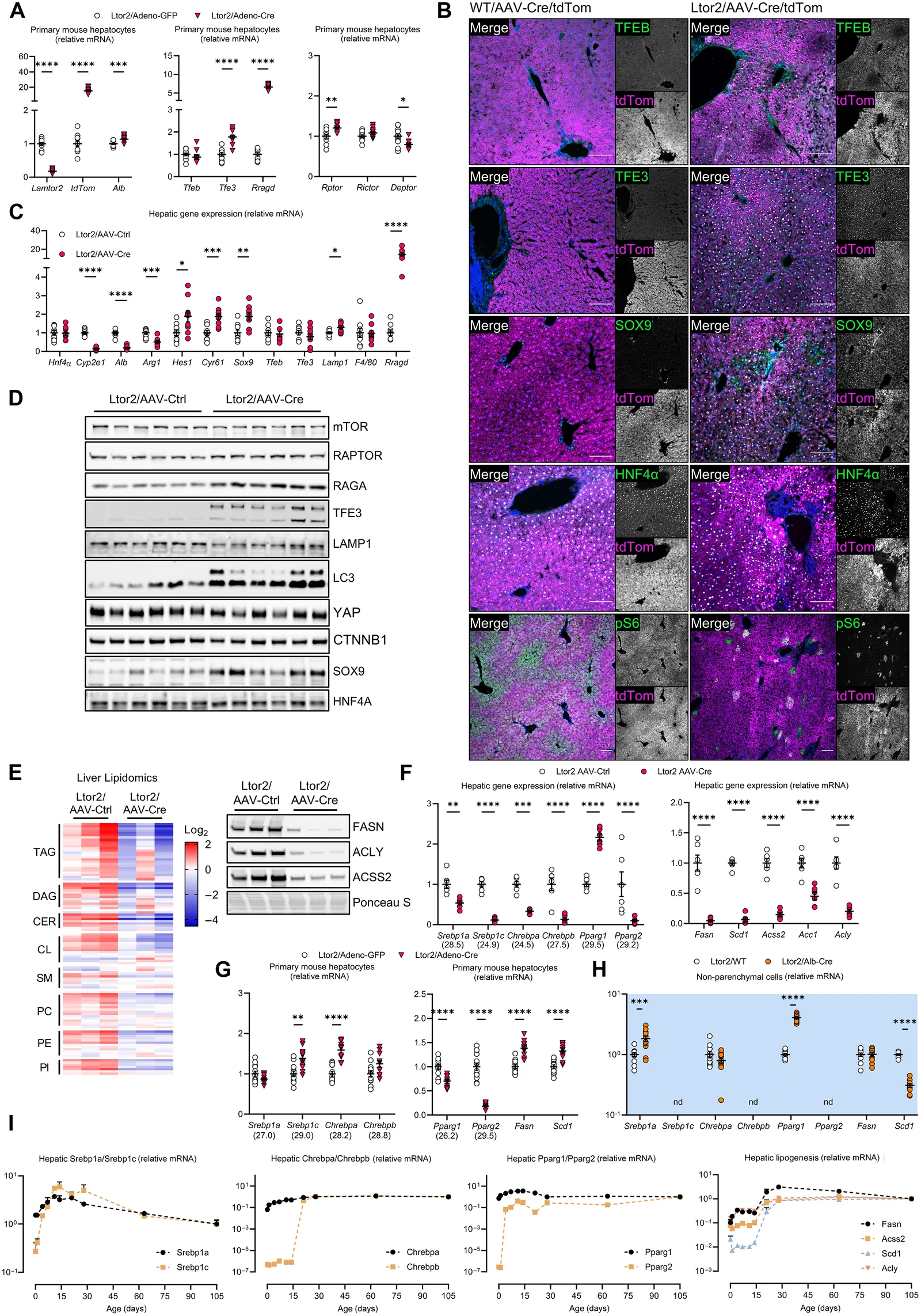
Lamtor2-deficient hepatocytes reprogram toward an early postnatal-like metabolic state. (**A**) mRNA expression of hepatobiliary marker genes and genes related to canonical and noncanonical mTORC1 signaling in primary mouse hepatocytes isolated from *Lamtor2*^flox/flox^ mice and treated in vitro with control or Adeno-CMV-Cre virus (*n* = 12 from at least two mice per condition). (**B**) Representative confocal images showing tdTomato with TFEB, TFE3, SOX9, HNF4α, or pS6 in Ltor2/AAV-Ctrl and Ltor2/AAV-Cre livers four weeks after virus injection. Images are representative of at least three male and female mice per condition. Scale bars, 100 μm. (**C** and **D**) Hepatic expression and immunoblot analysis of hepatobiliary and lysosome-associated markers in Ltor2/AAV-Ctrl and Ltor2/AAV-Cre mice (*n* = 6 to 9). (**E**) Hepatic lipid profiles measured by LC-MS and immunoblot analysis of lipogenic proteins in the same mice (*n* = 3). Lipid species were normalized to the average of all mice. (**F**) Hepatic expression of lipogenic genes and TF isoforms in male Ltor2/AAV-Ctrl and Ltor2/AAV-Cre mice (*n* = 6). (**G**) Expression of lipogenic genes and TF isoforms in control- and Cre-treated *Lamtor2*^flox/flox^ primary hepatocytes (*n* = 12 from at least two mice per condition). For (F) and (G), mean Ct values of the control group are shown in parentheses below selected transcript names. (**H**) Expression of lipogenic genes and TF isoforms in nonparenchymal cells from control and Ltor2-LKO mice (*n* = 12 from at least two mice per condition). (**I**) Developmental expression of lipogenic genes and TF isoforms from postnatal days 1 to 105 (*n* = 3 to 5 per time point). nd, not detectable. Data are means ± SEM. Statistical significance was calculated by two-tailed unpaired Student’s *t* test [(A), (C), (F), (G), and (H)]. *P < 0.05, **P < 0.01, ***P < 0.001, and ****P < 0.0001.

Hepatocytes establish specific metabolic functions with maturation, showing reciprocal expression of gene programs related to proliferation/differentiation and cholesterol, BA, and fatty acid metabolism (*24*). The loss of a functional Ragulator/mTORC1 signaling complex caused marked suppression of ATP-citrate-lyase (*Acly*), fatty acid synthase (*Fasn*), acyl-CoA synthetase short chain family member 2 (*Acss2*), accompanied by reduced lipid species derived from DNL and reduced total hepatic lipid content (Fig. 3, E and F). Thus, despite the clear hepatocellular appearance observed histologically, Ltor2/AAV-Cre livers did not show increased hepatic lipid accumulation in this analysis. SREBP1, CHREBP, or PPARG are predominant transcriptional regulators of DNL in the liver. These TFs share genomic organization that permits isoform-specific expression through 5′ alternative promoter usage and differential splicing. In the liver, Srebp1c is 10 times more abundant than Srebp1a, whereas Srebp1a or Pparg1 are more abundant in macrophages and mesenchymal cells (*25*–*27*). In Ltor2/AAV-Cre livers, *Lamtor2* deletion altered the expression pattern of lipogenic TF isoforms, with relative enrichment of Srebp1a, Chrebpa, and Pparg1 compared with their corresponding alternative isoforms (Fig. 3F). By contrast, immediate in vitro knockout (KO) by Adeno-Cre did not reproduce this pattern (Fig. 3G). The expression pattern of lipogenic TF isoforms was reminiscent of NPCs (Fig. 3H and fig. S4D) and of juvenile livers during the first two postnatal weeks of hepatocyte maturation (Fig. 3I and fig. S4E). However, Chrebpβ should be interpreted cautiously, as its expression peak around day 21 may also reflect the dietary transition to carbohydrate-rich chow after weaning. In contrast, *Lamtor2*, *Tfe3*, and *Tfeb* as well as the expression of fasting/oxidative genes (*Fgf21*, *Igfbp1*, *Cpt1a*, and *Cpt1b*) were higher at birth and remained unchanged or declined from day 14 onward (fig. S4E).

Recent studies showed that the loss of hepatic FLCN using AAV8-Cre results in reduced DNL due to forced nuclear TFE3 expression. However, in contrast to Ltor2/AAV-Cre, these animals maintain normal body weight and liver histology (*18*), suggesting that preserved canonical mTORC1 signaling may compensate for alterations in liver pathology. Mice lacking the regulatory-associated protein of mTOR (RAPTOR) in adult hepatocytes (Rptor/AAV-Cre) showed similar alterations in liver histology and serum biochemistry as Ltor2/AAV-Cre animals (fig. S5, A to E), with reduced serum glucose, and suppressed hepatic DNL (fig. S5, F to H), but elevated hepatic TAGs (fig. S5I), which is due to reduced very-low-density lipoprotein (VLDL) secretion (*20*). Gene expression was similarly altered, with decreased hepatocyte and increased progenitor-like genes, nuclear TFE3 retention, and higher *Rragd* expression (fig. S5, J to M). Notably, hepatocyte foci with persisting mTORC1 activity (∼5% pS6 at 2 weeks after AAV-Cre) expanded and formed multicellular foci (∼50% pS6-positive cells after 6 weeks of AAV-Cre), which remained limited in Ltor2/AAV (fig. S5, N and O). Persistent phosphorylation of S6 in Ltor2/AAV or Rptor/AAV was not accompanied by an obvious increase in hepatocyte Ki67 staining under the conditions examined (fig. S5P) and did not appear to reflect compensatory activation of other signaling pathways, consistent with previous observations in Rag-deficient cells or animals (*28*). Moreover, additional liver injury induced by 0.5% cholic acid (CA) markedly increased Ki67 staining but did not cause an overt expansion of pS6-positive hepatocytes in Rptor/AAV or WT livers (fig. S5Q). Together, these data do not support a simple association between the pS6-positive foci and hepatocyte proliferation after liver injury under the conditions examined. To determine whether pS6-positive hepatocyte subpopulations can also arise in response to PC-restricted injury, we examined livers from acetaminophen (APAP)–treated animals, a model in which ANXA2-positive injury–associated/interface hepatocytes emerge at the regenerating interface (*29*, *30*). In this qualitative analysis, interface hepatocytes showed pS6 staining that was most evident at 36 hours after APAP treatment (fig. S6, A to C). These interface hepatocytes in PC regions, similar to cholangiocytes in PP areas, also stained positive for SOX9. APAP-treated livers further showed mixed portal and central phosphoenolpyruvate carboxykinase and cholesterol 7 alpha-hydroxylase (CYP7A1) expression, which, similar to pS6, extended into broader areas at 36 and 48 hours (fig. S6D). Portal-central polarity, SOX9, and pS6 expression were largely restored by 96 hours after APAP treatment (fig. S6, C and D). Together, these findings support a role for the mTORC1/TFEB-TFE3 axis in hepatocyte plasticity and metabolic identity in the genetic models studied here.

### Loss of Lamtor2 alters BA metabolism and activation of FXR

The liver controls several metabolic functions including lipid and glucose, cholesterol, and BA homeostasis. During postnatal development, the liver biosynthetic capacity for BAs increases, suggesting that hepatic BA synthesis is another feature of hepatocyte maturation (*31*). The BA pool consists of 12-hydroxylated (12-OH) BAs [such as CA and deoxycholic acid (DCA)] and 6-hydroxylated (6-OH) BAs [such as muricholates (MCAs), murideoxycholic acid (MDCA), hyocholic acid (HCA), and hyodeoxycholic acid (HDCA)]. Non–12- and non–6-hydroxylated BAs, such as [chenodeoxycholic acid (CDCA), ursodeoxycholic acid (UDCA), and lithocholic acid (LCA)] are less abundant in rodents but occur naturally in humans. In contrast to WT mice, CDCA was a predominant BA in Ltor2/AAV mice (Fig. 4, A to E, and fig. S7A), causing a decrease in 12-OH/non–12-OH and 6-OH/non–6-OH ratios. Elevated unconjugated BAs (UBAs) contrasted with the normally, almost exclusively conjugated biliary BAs (CBAs) (Fig. 4C). Expression levels of BA biosynthesis genes and enzymes were virtually absent, except for CYP7A1 (Fig. 4, F and K, and fig. S7, B to D). Changes in BA composition known to compromise lipid absorption and/or lipoprotein secretion due to low 12-OH/non–12-OH and enrichment of biliary CDCA (*20*, *32*) were associated with low plasma TAG and cholesterol levels (fig. S7, E to J). Advanced imaging analysis confirmed hepatic enrichment of dihydroxy BAs (most likely TCDCA; Fig. 4, A and D) and a decrease of trihydroxy BAs (fig. S7K). Ultrastructural analysis with three-dimensional (3D) organelle reconstruction demonstrated dilated endoplasmic reticulum in Ltor2/AAV hepatocytes, particularly at mitochondrial contact sites (fig. S7L), with rough-ER curving and wrapping around lysosomes and mitochondria (fig. S7M). These findings suggest altered organelle communication in *Lamtor2*-deficient hepatocytes and are in line with emerging links between organelle-contact remodeling and hepatocyte dedifferentiation (*33*).

**Fig. 4.**
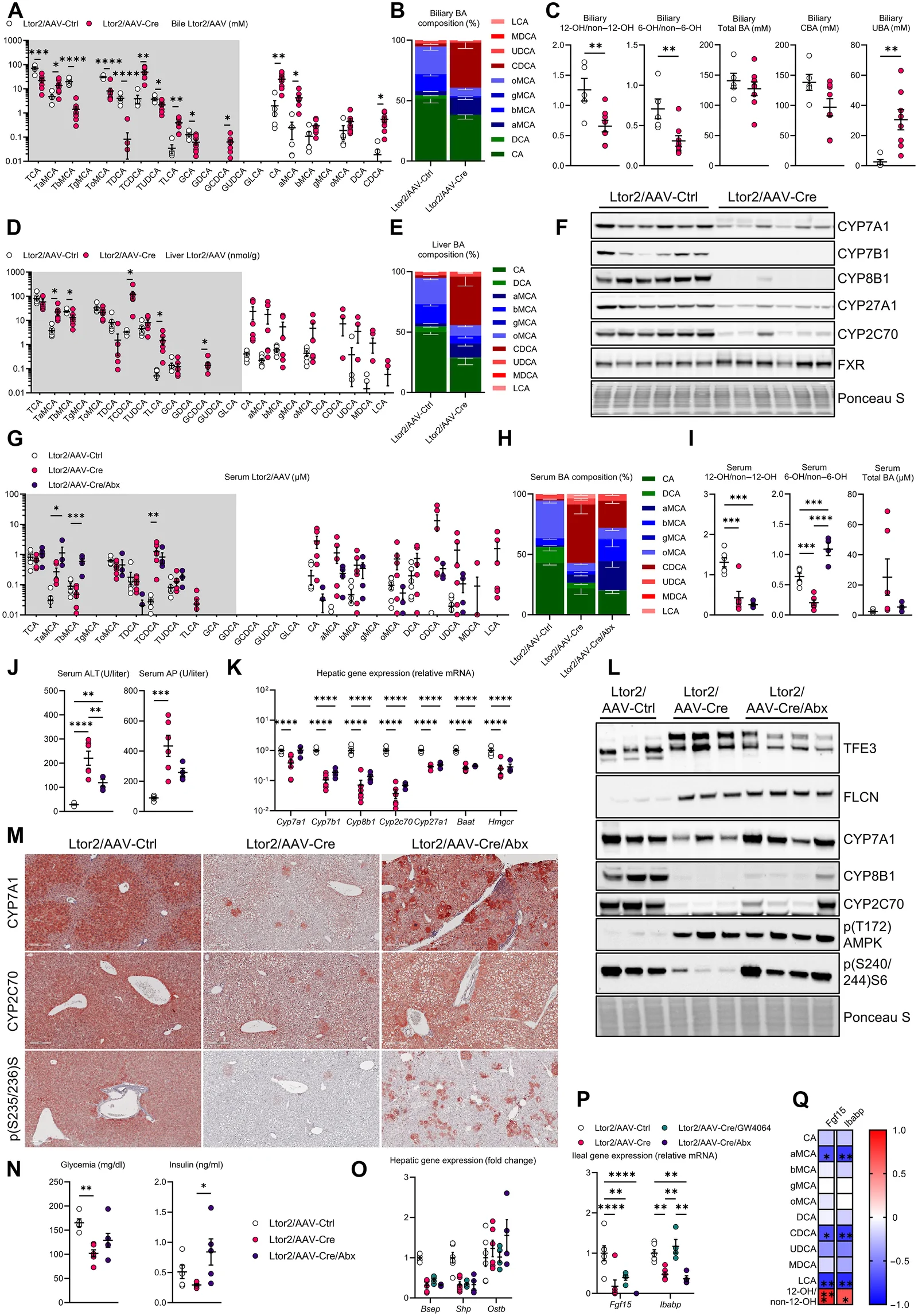
Lamtor2 deletion alters BA metabolism, composition, and signaling. (**A** to **E**) BA profiling in bile and liver from male Ltor2/AAV-Ctrl and Ltor2/AAV-Cre mice four weeks after virus injection (*n* = 5 to 8). (A) Absolute BA levels in bile. (B) Biliary BA composition. (C) Biliary 12-OH/non–12-OH and 6-OH/non–6-OH ratios, total BAs, conjugated BAs, and UBAs. [(D) and (E)] Absolute BA levels and BA composition in liver. (**F**) Immunoblot analysis of BA biosynthetic enzymes and FXR in liver extracts from Ltor2/AAV-Ctrl and Ltor2/AAV-Cre mice. (**G** to **J**) Serum BA levels, composition, 12-OH/non–12-OH and 6-OH/non–6-OH ratios, total BAs, ALT, and AP in Ltor2/AAV-Ctrl, Ltor2/AAV-Cre, and antibiotic-treated Ltor2/AAV-Cre mice (*n* = 4 to 6). Antibiotic treatment was given for the final two weeks before analysis. (**K** and **L**) Hepatic expression of BA/cholesterol metabolism genes and immunoblot analysis of TFE3, FLCN, CYP7A1, CYP8B1, CYP2C70, pAMPK, and pS6 in the same groups (*n* = 3 to 6). (**M**) Representative liver sections stained for CYP7A1, CYP2C70, and pS6. Scale bars, 200 μm. (**N**) Serum glucose and insulin in the same treatment groups (*n* = 5 to 6). (**O** and **P**) Hepatic and ileal FXR target-gene expression in Ltor2/AAV-Ctrl, Ltor2/AAV-Cre, GW4064-treated Ltor2/AAV-Cre, and antibiotic-treated Ltor2/AAV-Cre mice (*n* = 4 to 6). (**Q**) Spearman correlation analysis between ileal Fgf15/Ibabp expression and serum BA species in male and female Ltor2/AAV-Ctrl and Ltor2/AAV-Cre mice (*n* = 12). Data are means ± SEM. Statistical significance was calculated by two-tailed unpaired Student’s *t* test [(A), (C), and (D)] or one-way analysis of variance (ANOVA) with Holm-Šídák post hoc testing [(G), (I) to (K), and (N) to (P)]. *P < 0.05, **P < 0.01, ***P < 0.001, and ****P < 0.0001.

Microbiota-mediated changes in BA composition regulate host metabolism through the BA receptor farnesoid X receptor (FXR). Depletion of the gut microbiome reduces BA transformation by decreasing UBAs and enriching the BA-pool with 6-OH CBAs (*34*). In Ltor2/AAV-Cre mice, antibiotic treatment (Abx) decreased major bacterial BAs (DCA and LCA), lowered UBAs (Fig. 4, G to I), and significantly improved liver injury in association with restored conversion of CDCA to 6-OH MCAs (Fig. 4, G to J). Abx treatment also partially restored expression of BA synthesis enzymes, reactivated canonical mTORC1 (pS6) and normalized TFE3 expression without affecting elevated FLCN or pAMPK levels (Fig. 4, K and L). Representative imaging showed that the reexpression of BA enzymes coincided with newly appearing pS6-positive hepatocyte clusters (Fig. 4M). Notably, Abx was also associated with altered systemic glucose/insulin parameters, including increased circulating insulin relative to untreated Ltor2/AAV-Cre mice, which may have contributed to the reappearance of pS6-positive hepatocyte clusters (Fig. 4N).

Because Abx altered both BA composition and systemic insulin signaling, we next asked whether mTORC1 activity could also influence FXR responsiveness. BAs act as endocrine hormones to coordinately regulate hepatic metabolism through activation of FXR (*8*). In Ltor2/AAV mice, FXR signaling was severely compromised and even administration of GW4064, a potent FXR agonist, resulted only in modest activation of FXR target genes in liver and intestine (Fig. 4, O and P). The expression of Fgf15 and Ibabp correlated negatively with aMCA, CDCA, and LCA but positively with the 12-OH/non–12-OH ratio (Fig. 4Q), suggesting that Ltor2/AAV mice are unable to produce major FXR-agonistic BAs. However, although CDCA is a potent FXR agonist, at least in humans (*35*), diminished induction of FXR target genes by GW4064 suggested that mTORC1 may also affect FXR responsiveness. To test this more directly, we treated human hepatoma cells (HepG2) with Torin1, which reduced pS6 signaling (fig. S8A) and promotes nuclear TFEB/TFE3 localization. Torin1 treatment was associated with positive enrichment of gene sets such as epithelial-mesenchymal transition (EMT) and negative enrichment of mTORC1 signaling, cholesterol, fatty acid, and BA metabolism gene sets (fig. S8, B and C). Notably, Torin1 strongly attenuated the induction of FXR target genes by GW4064 and CDCA in HepG2 cells, as well as in Huh7 liver and HT29 intestinal cell lines (fig. S8, B and D). These findings are consistent with previous in vivo data showing that loss of *Flcn* and activation of TFE3 suppress lipogenic programs linked to nuclear receptor signaling (*18*). Thus, mTORC1 appears to influence BA metabolism and lipid homeostasis, at least in part through effects on nuclear hormone receptor signaling.

### TFEB/TFE3-associated feedback signaling contributes to mTORC1-dependent BA homeostasis

Because previous work has shown that MiT/TFE TFs can feed back onto canonical mTORC1 signaling through Ragulator/Rag pathway components, including *Rragd*/RRAGD, we next asked whether altered TFEB/TFE3 activity is associated with changes in canonical mTORC1 signaling and BA metabolism in vivo (*36*, *37*). To distinguish the consequences of Ragulator loss and canonical mTORC1 suppression from those of MiT/TFE activation under preserved or hyperactive canonical mTORC1 signaling, we next examined complementary genetic models targeting distinct nodes of this pathway. Thus, these models were used to separate signaling states rather than to provide redundant confirmation of the same phenotype. We first tested whether reducing TFEB/TFE3 activity modifies the *Lamtor2*-deficient phenotype and then compared this with models of preserved or hyperactive canonical mTORC1 signaling. Because TFE3 and TFEB have cooperative and partially redundant functions in energy metabolism (*38*), we induced *Lamtor2* deficiency in *Tfeb*- (BKO), *Tfe3*- (3KO), and combined *Tfeb*/*Tfe3*-deficient mice [double KO (DKO)] using an AAV8-CRISPR-Cas9 strategy.

However, viral CRISPR-Cas9 reduced LAMTOR2 protein levels by about 40 to 50% in BKO and ∼80 to 90% in 3KO and DKO livers, likely reflecting incomplete editing and/or tissue heterogeneity in bulk liver extracts. We therefore do not interpret residual LAMTOR2 as reexpression in fully edited KO cells, but as compatible with higher LAMTOR2 levels in incompletely edited or nontargeted cells. In line with a TFEB/TFE3-linked feedback model, previous work has shown that MiT/TFE factors can bind CLEAR motifs in promoters of mTORC1-related genes, including Rrag and Lamtor family members (*36*). Consistent with this, *Rragd* expression increased after *Lamtor2* knockdown, and this induction was blunted by additional *Tfe3* KO or combined *Tfeb*/*Tfe3* KO (fig. S9, A and B). In this setting, elevated 6-OH/non–6-OH and reduced 12-OH/non–12-OH BA ratios and expression of BA biosynthesis enzymes were almost unaffected by additional loss of BKO or 3KO (fig. S9, A to D). By contrast, virus-mediated TFEB overexpression in the liver of WT mice leads to increased mTORC1 signaling, which was associated with increased 12-OH and decreased 6-OH BAs (fig. S9, E and F). These findings suggest that cross-talk between canonical mTORC1 signaling and TFEB/TFE3 drives alterations in BA metabolism. In this context, previous studies have shown that MiT/TFE factors can promote mTORC1 activity through transcriptional feedback mechanisms involving Rag pathway components (*36*, *37*). We therefore examined BA metabolism in liver-specific *Flcn* KO (FKO) mice with forced nuclear TFE3 expression and preserved/active canonical mTORC1 (*18*). Loss of FLCN (FKO) resulted in increased 12-OH and decreased 6-OH BA ratios, which contrasts our findings observed in *Lamtor2* and *Raptor* KO animals, with high TFEB/TFE3 activity. Conversely, *Tfe3* KO mice (3KO) showed high levels of CYP2C70 and low expression of CYP8B1 resulting in elevated 6-OH and diminished 12-OH BA ratios and thereby reverted abnormalities of serum BA composition as well as high pS6 and LAMTOR2 expression levels when crossbred with FKO mice (Fig. 5, A to D). Moreover, the loss of mTORC1 signaling in Rptor/AAV-Cre mice resulted in reduced 12-OH BAs and enrichment for CDCA as well as 6-OH BAs, while gain of mTORC1 activity in Tsc1/AAV-Cre mice increased 12-OH/non–12-OH and lowered 6-OH BAs (Fig. 5, F and G) despite TFEB/TFE3 activity in both models. Since 12α-hydroxylated BA species are enriched in MASLD and MASH (*11*–*15*), these findings may be relevant to metabolic liver disease.

**Fig. 5.**
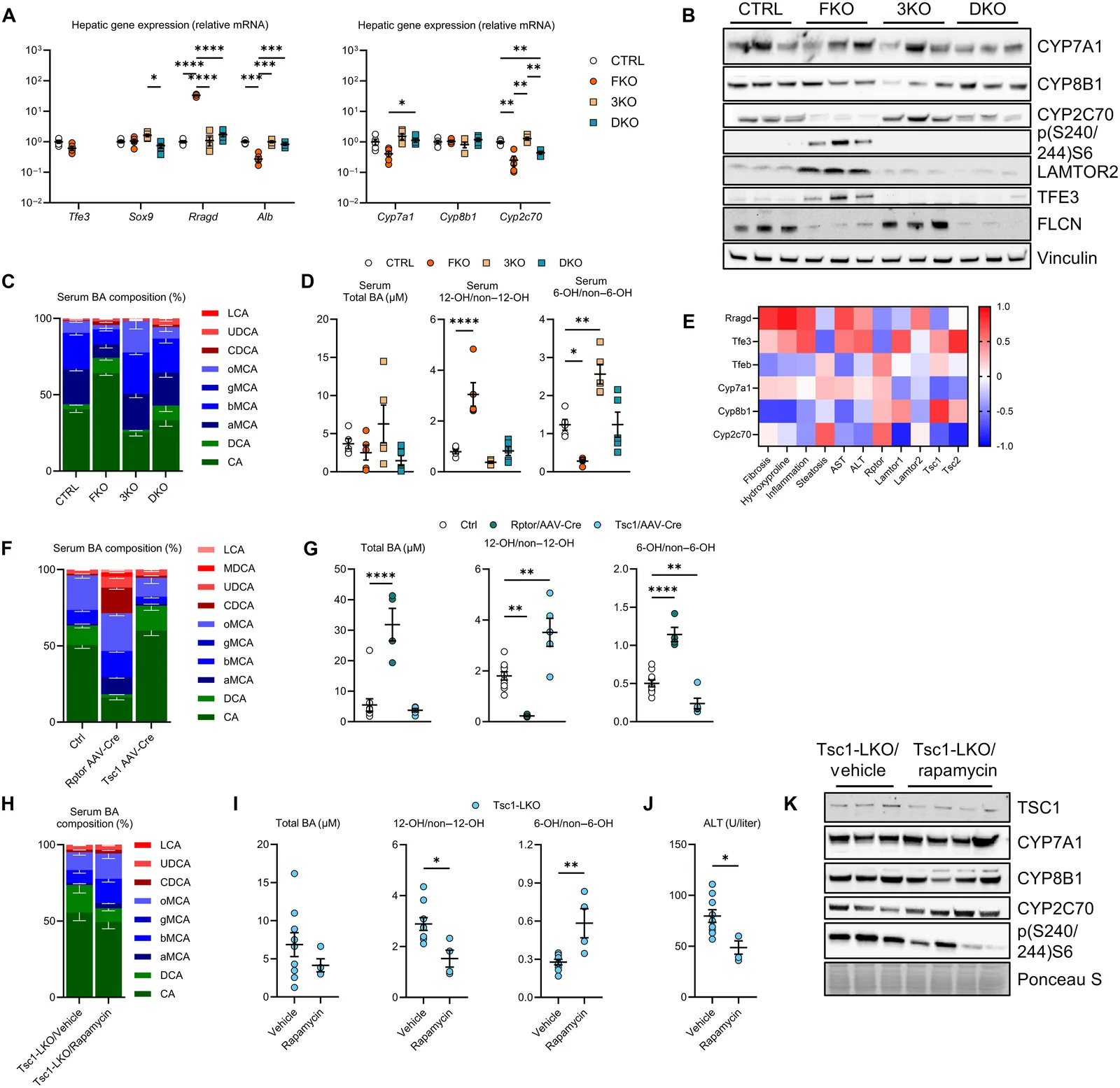
Canonical mTORC1 signaling modulates BA homeostasis across complementary genetic models. (**A** and **B**) Hepatic mRNA expression of hepatobiliary markers, non-canonical mTORC1 targets, and BA biosynthetic enzymes, and immunoblot analysis of BA biosynthetic proteins, pS6, and LAMTOR2 in control, Flcn-LKO (FKO), Tfe3-KO (3KO), and DKO mice (*n* = 3 to 5). (**C** and **D**) Serum BA composition, 12-OH/non–12-OH and 6-OH/non–6-OH ratios, and total BAs in the same genotypes (*n* = 5). (**E**) Heatmap showing Spearman correlations between hepatic *Rragd*, *Tfe3*, *Tfeb*, *Cyp7a1*, *Cyp8b1*, and *Cyp2c70* expression and the indicated parameters in fructose, palmitate, and cholesterol (FPC) diet and choline-deficient, methionine-reduced high-fat diet (CDAHFD) mouse MASH datasets. (**F** and **G**) Serum BA composition, 12-OH/non–12-OH and 6-OH/non–6-OH ratios, and total BAs in Rptor/AAV-Cre, Tsc1/AAV-Cre, and control mice four weeks after virus injection (*n* = 4 to 11). (**H** to **J**) Serum BA composition, 12-OH/non–12-OH and 6-OH/non–6-OH ratios, total BAs, and serum ALT in vehicle-treated Tsc1-LKO mice, and rapamycin-treated Tsc1-LKO mice (*n* = 4 to 9). (**K**) Immunoblot analysis of TSC1, BA biosynthetic enzymes, and pS6 in vehicle- and rapamycin-treated Tsc1-LKO livers. Data are means ± SEM. Statistical significance was calculated by two-tailed unpaired Student’s *t* test [(I) and (J)] or one-way ANOVA with Holm-Šídák post hoc testing [(A), (D), and (G)]. *P < 0.05, **P < 0.01, ***P < 0.001, and ****P < 0.0001.

We mined data sets from murine MASH models (*39*) and correlated genes of the Ragulator/mTORC1 pathways and BA synthesis genes with MASLD/MASH parameters, such as fibrosis, inflammation, steatosis, and liver damage (ALT and AST). *Rragd* highly correlated with liver fibrosis and inflammation, while *Cyp8b1* was anticorrelated with liver injury and fibrosis. Particularly, Tsc1/2 expression correlated positively with *Cyp8b1* and inversely with *Cyp2c70* (Fig. 5E), indicating that constitutively active/hyperactive mTORC1 drives 12-OH BA enrichment. Short-term rapamycin treatment of Tsc1-LKO mice reduced liver injury and shifted the BA profile toward lower 12-OH/non–12-OH and higher 6-OH/non–6-OH ratios, accompanied by reduced CYP8B1 and increased CYP2C70 expression (Fig. 5, H to K, and fig. S10, A and B). Together, these data identify divergent responses to canonical mTORC1 signalingand support a model in which TFEB/TFE3-linked feedback maintains either 12-OH or non–12-OH BA synthesis depending on whether canonical mTORC1 output is suppressed, preserved, or hyperactive. Hyperactive mTORC1 may therefore contribute to MASLD and MASH pathologies via synthesis of specific BA species (*18*–*20*).

### mTORC1 shapes the landscape of BAs associated with hallmarks of MASLD pathology

Our observations suggested that loss and gain of functional mTORC1 signaling could contribute to BA alterations during dietary induced MASLD development. We thus administered mice to MASLD-inducing diets for 12 weeks but performed experiments using Alb-Cre mice (fig. S10, A to C), as AAV8-Cre deletion of *Raptor* showed compensatory canonical mTORC1 activation over time (fig. S5N). Rptor-LKO and Tsc1-LKO showed comparable changes in BA pool composition as compared to AAV-Cre mice (fig. S10, D to K). On a high cholesterol, high fat diet without sucrose (HCHF), Tsc1-LKO mice developed PP macrovesicular steatosis, while Rptor-LKO developed microvesicular steatosis with only few larger LDs as present in controls (Fig. 6A). The same diet, enriched in sucrose (HSHCHF) led to macrovesicular steatosis in both Tsc1-LKO and in Rptor-LKO, which contrasts the PC microvesicular in WT animals. Lipidomics analysis revealed that mice on HSHCHF diet accumulated more TAGs, with increased fatty acid chain length and polyunsaturation, as compared to HCHF, which was most pronounced in Tsc1-LKO mice and resulted in slightly lower TAG species in Rptor-LKO livers compared to WT controls (Fig. 6, B and C). Liver injury was significantly elevated in mice fed HSHCHF diet, regardless of genotype (Fig. 6, D and E). Body weight gain on both diets was attenuated in Rptor-LKO and Tsc1-LKO animals (Fig. 6, F and J), without significantly altering ad libitum serum glucose or insulin levels (Fig. 6, G and K). Glucose tolerance tests (GTTs) in Rptor-LKO mice with higher levels of 6-OH-BAs showed significantly improved glucose sensitivity, whereas Tsc1-LKO mice with elevated 12-OH-BAs showed a significantly reduced glucose tolerance response (Fig. 6, H and I, L, and M). The delivery of cholesterol from endocytic carriers toward mitochondria serves as a substrate for steroid hormone synthesis (*40*). The localization of free cholesterol with the mitochondrial marker OPA1 on super high-resolution microscopy was less pronounced in Tsc1-LKO mice as compared with Rptor-LKO and control animals (Fig. 6, N and O). Our data therefore indicate that transport of dietary cholesterol and cholesterol trafficking (*41*, *42*) could be part of the mechanism by which mTORC1 activity shapes BA composition toward 12-OH or 6-OH BAs.

**Fig. 6.**
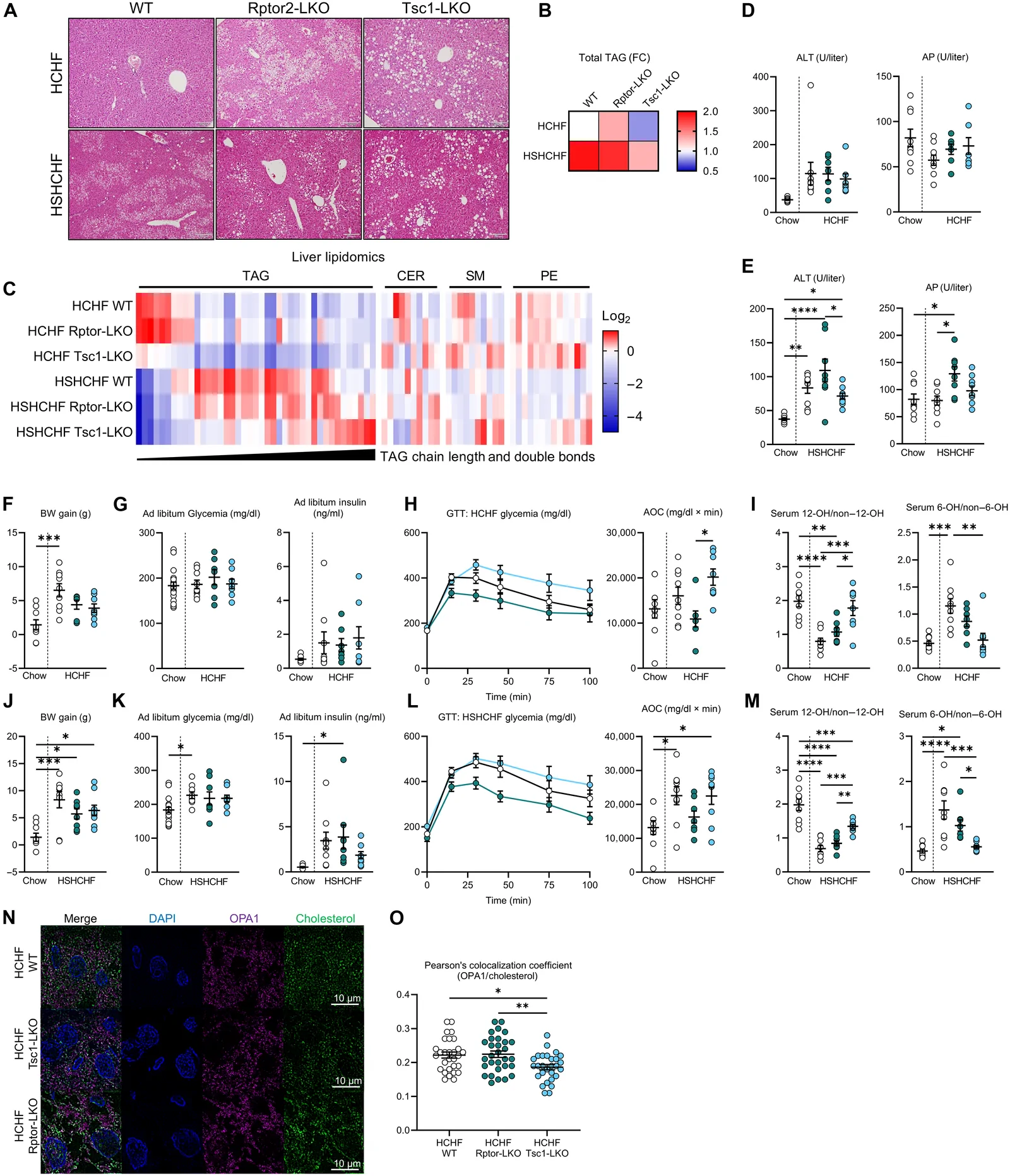
mTORC1 shapes BA profiles associated with MASLD pathology. (**A** to **M**) WT, Rptor-LKO, and Tsc1-LKO mice were fed a high-cholesterol/high-fat diet (HCHF) or high-sucrose/high-cholesterol/high-fat diet (HSHCHF) for 12 weeks (*n* = 7 to 9). (A) Representative H&E-stained liver sections. Scale bars, 200 μm. (B) Hepatic TAGs measured by LC-MS and shown relative to HCHF-fed control mice. (C) Hepatic lipid profiles measured by LC-MS. Each lipid species was normalized to the average of all mice (*n* = 3). [(D) and (E)] Serum ALT and AP in HCHF- and HSHCHF-fed mice. [(F) to (I)] Body-weight gain, serum glucose/insulin, glucose tolerance, and serum 12-OH/non–12-OH and 6-OH/non–6-OH BA ratios after HCHF feeding. [(J) to (M)] Body-weight gain, serum glucose/insulin, glucose tolerance, and serum 12-OH/non–12-OH and 6-OH/non–6-OH bile acid ratios after HSHCHF feeding. (**N**) Representative high-resolution images of free cholesterol (Filipin) and the mitochondrial marker OPA1 in livers from HCHF-fed WT, Rptor-LKO, and Tsc1-LKO mice. (**O**) Quantification of Filipin and OPA1 colocalization. Data are means ± SEM. Statistical significance was calculated by one-way ANOVA with Holm-Šídák post hoc testing [(D) to (M) and (O)]. *P < 0.05, **P < 0.01, ***P < 0.001, and ****P < 0.0001.

CYP8B1 haploinsufficiency in humans is associated with increased insulin sensitivity (*10*), and elevated 6-OH BAs in *Cyp8b1* KO mice protect against obesity and IR induced by a high-fat diet (HFD). Consistent with previous work, hepatic mTORC1 activation in the setting of impaired insulin action suppresses mitochondrial oxidative metabolism and contributes to hyperglycemia (*43*), while AKT/mTORC1 signaling also regulates hepatic SREBP1c-dependent lipogenesis (*44*). In our models, hyperactive canonical mTORC1 was additionally associated with preferential enrichment of 12-OH BAs. Our CRISPR-Cas9–mediated *Cyp8b1* knockdown in Tsc1-LKO mice decreased serum TAG and cholesterol levels (fig. S11, A to D). The increase in 6-OH BAs was only modest (fig. S11, B and C), and CYP8B1 protein expression was reduced by only ∼40% as compared to control animals (fig. S11A), suggesting that constitutive mTORC1 activation may help preserve CYP8B1 protein expression in Tsc1-LKO livers by regulation of mRNA/protein stability (fig. S10C). This would be consistent with broader evidence that mTORC1 can regulate hepatic metabolic programs through posttranscriptional mechanisms (*45*). We next determined if dietary protein restriction (PR), which leads to mTORC1 inactivation (*46*), would mimic BA compositions observed in Rptor-LKO mice. PR led to a tremendous reduction of CYP8B1, but not CYP7A1, decreasing 12-OH BAs with concomitant enrichment of 6-OH BAs but, somewhat similar to our foregoing CRISPR-Cas9 experiment, was unable to reduce CYP8B1 expression in Tsc1-LKO livers (fig. S11, E to J). Notably, this led to persistently higher 12-OH BAs in Tsc1-LKO mice as compared to control mice on PR diet (fig. S11, I and J). Our data suggest that the inability to lower hyperactive mTORC1 in Tsc1-LKO mice, either by PR or during feeding-fasting cycles, contributes to persistently elevated *Cyp8b1* expression levels (fig. S11, G and K and L). In patients with MASLD and MASH, we positively correlated serum CA levels with markers of liver injury (AP and GGT) and inversely with TSC1 expression (fig. S12A). CYP8B1 expression was higher in MASLD and MASH liver samples as compared to CYP7A1, while TSC1 levels tended to be higher, especially in patients with MASH (fig. S12B). We next investigated whether a low-protein (LP) diet, similar to our findings in mice, would alter BA metabolism in patients with MASLD. This LP-dietary intervention was previously shown to reduce BMI, fasting glucose, serum insulin, and HOMA-IR levels (*47*). Serum BA analysis demonstrated decreased serum CA (12-OH), and increased non–12-OH BAs, mainly CDCA (fig. S12C). The LP diet also reduced CYP8B1 expression in liver biopsies, while CYP7A1 expression remained unchanged (fig. S12D). Patients on a high-protein (HP) diet had unchanged 12-OH/non–12-OH BA ratios and showed equal staining of both CYP7A1 and CYP8B1 (fig. S12D). Together, our results indicate that constitutive mTORC1 activity drives alterations in the BA pool, enriching for 12-OH BAs, which are associated with diabetes and IR in both rodents and MASLD/MASH patients. Dietary PR might therefore be a therapeutic option for diseases with dysfunctional metabolism linked to altered (“unfavorable”) BA compositions.

## DISCUSSION

Our study underscores the critical role of Ragulator-Rag GTPase components in MiT/TFE-linked mTORC1 feedback signaling, which is associated with convergent and divergent regulation of hepatocyte plasticity, lipid metabolism, and BA homeostasis. Specifically, the hepatic deletion of *Lamtor2* impaired hepatocyte-specific gene expression and metabolic pathways related to lipid, cholesterol, and BA metabolism, resembling transcriptional profiles seen during murine postnatal liver development. These findings support the concept that Ragulator/mTORC1 signaling contributes to the maintenance of hepatocyte metabolic identity.

However, the developmental comparison should be interpreted with caution. Postnatal nutritional transitions, particularly the shift from milk to chow during weaning, may contribute to some of the observed gene-expression trajectories. This may be especially relevant for genes such as Chrebpβ, which change more abruptly around the weaning period, whereas the more gradual trajectories of other isoforms are also consistent with intrinsic hepatocyte maturation programs. In *Lamtor2*-deficient livers, these changes were accompanied by features of altered lineage specification; persistent nuclear localization of TFEB/TFE3; and altered SREBP1, CHREBP, and PPARG isoform patterns resembling those observed in nonparenchymal or developing liver cells (*48*).

Accordingly, the comparison across *Lamtor2*-, *Raptor*-, *Flcn*-, and *Tsc1*-deficient models was intended to distinguish BA phenotypes linked to suppression of canonical mTORC1 from those observed when MiT/TFE activity occurs in the presence of preserved or hyperactive canonical mTORC1 signaling. Because *Lamtor2* and *Raptor* deficiency also impair broader hepatocyte identity and liver function, BA remodeling in these models should be interpreted in the context of this altered hepatocellular state rather than as a purely isolated BA-specific module. Our data reveal that an elaborate cross-talk between mTORC1 and TFEB/TFE3 orchestrates the synthesis of distinct BA species, which may partly explain phenotypic divergences of loss and gain of functional mTORC1 in genetic mouse models. Constitutive active TFEB/TFE3 in FKO and Tsc1-LKO mice (*36*, *37*) drives elevated 12-OH BAs through suppression of *Cyp2c70* (reduced 6-OH BAs) and activation of *Cyp8b1* (increased 12-OH BAs). We also found strong correlation between Tsc1/2 expression and *Cyp8b1* in murine MASH models and between serum 12-OH BA levels in human MASLD. Elevated 12-OH BAs have been attributed to IR and FOXO1 activation. Prior studies have linked insulin resistance and FOXO1 activity to increased 12α-hydroxylated BA synthesis through regulation of *Cyp8b1* (*9*, *49*, *50*). Although this may appear to contrast with our finding that hyperactive canonical mTORC1 states are associated with increased 12-OH BAs, insulin-FOXO1 signaling and mTORC1 activity are not necessarily coupled in a linear manner. Liver insulin receptor KO mice show impaired BA synthesis and a lithogenic BA profile with a decreased muricholate-to-cholate ratio, indicating that isolated hepatic insulin resistance does not simply phenocopy *Lamtor2*- or *Raptor*-deficient models (*51*). Our data therefore suggest that BA output depends on the broader signaling state, with low canonical mTORC1/high TFEB-TFE3 activity favoring non–12-OH/6-OH BA enrichment and preserved or hyperactive canonical mTORC1 favoring 12-OH BA enrichment. This context-dependent view is further supported by recent work linking HSDL2 to PGC1α-PPARα and PI3K-mTORC1–dependent regulation of BA and cholesterol homeostasis (*16*). In line with this interpretation, FOXO1 disinhibition alone only modestly increases *Cyp8b1* (*50*, *51*), and hepatic FOXO1 deletion marginally decreases 12-OH BAs (*9*), suggesting compensatory adaptation, possibly by chronic Alb-Cre–mediated deletions in previous studies and highlighting that additional pathways cooperate with insulin-FOXO1 signaling to shape the BA pool. For instance, PPAR signaling, glucose, and steroid metabolism genes—including *Cyp2c70*, *Ppargc1a*, and *Lpin1*—were only affected when AAV-Cre was used (*9*, *52*). Thus, our data do not argue against insulin-FOXO1 regulation of *Cyp8b1* but suggest that this pathway intersects with mTORC1/MiT-TFE signaling and hepatocyte state in a context-dependent manner.

Feeding induces cholesterol biosynthesis via mTORC1 and dysregulated cholesterol metabolism, and cholesterol gallstone formation via 12-OH BAs during hepatic IR (*51*) has been associated with MASLD progression (*11*, *53*). Our data indicate that cholesterol-mTORC1 signaling (*17*, *54*) shapes the BA pool by integrating mitochondrial cholesterol transport (*40*, *41*) and de novo synthesis (*55*), pathways differentially regulated in hypo- versus hyperactive mTORC1 states. This is consistent with observations that 12-OH BAs are preferentially derived from de novo cholesterol, whereas for non–12-OH-BAs dietary cholesterol is the preferred substrate (*56*–*58*). 12-OH BAs are endogenous FXR agonists and could mediate the protective effect on hepatic steatosis, inflammation, and fibrosis in Tsc1-LKO livers. Non–12-OH BAs in rodents act as FXR antagonist, particularly in the intestine, and by lowering lipid absorption may explain the protection of Rptor-LKO mice under HFD (*50*, *59*). This however contrasts TAG accumulation during fasting, which is due to elevated NEFA influx and diminished VLDL secretion (*20*). An important part of our study shows that PR, similar to genetic or pharmacological mTORC1 inhibition, reduces 12-OH/non–12-OH BA ratio in both mice in vivo and in patients with MASLD. Individuals with IR and obesity have higher CYP8B1 activity, but reversing IR does not appear to affect this BA ratio (*60*). Notably, *Cyp8b1* increases several fold in fasted rats and mice, but not in rabbits, which, similar to humans, express high basal *Cyp8b1* levels (*61*). These differences underscore species-specific variations in BA regulation via nutrients and AKT/FOXO1 pathways under varying mTORC1 activity states. In the transition from prediabetes to full IR (*62*), hepatic IR (*63*) acting through the AKT/FOXO1 axis may exert distinct effects depending on whether mTORC1 is hypo- or hyperactive (*19*, *64*). Particularly, hydrophilic non–12-OH BAs reduce serum lipid levels in the context of mTORC1 suppression (*65*), protect from obesity (*66*, *67*), and correlate with improved outcomes from dietary interventions and enhanced insulin sensitivity (*15*, *68*, *69*).

Our study has a number of limitations: First, in the *Lamtor2*- and *Raptor*-deficient models, BA remodeling occurs alongside broader alterations in hepatocyte identity and liver function. Thus, some components of the BA phenotype may arise in the context of this altered hepatocellular state rather than from an isolated BA-specific regulatory module. This limitation is particularly relevant for bulk RNA, protein, and metabolite analyses, which average across heterogeneous hepatocyte and NPC populations. However, the use of acute adult AAV8-TBG-Cre models and comparison across complementary genetic states—including *Lamtor2*, *Raptor*, *Flcn*, and *Tsc1* perturbation—support the interpretation that BA remodeling is also shaped by the underlying mTORC1/MiT-TFE signaling state. Second, we could not clarify the origin of pS6-positive hepatocyte subpopulations arising in *Lamtor2*-deficient and Rptor/AAV mice. TFE3 activates insulin signals (*70*) and thereby could stimulate a dynamic nonlysosomal mTORC1 fraction that was recently shown to remain active toward S6K (*71*, *72*). RAPTOR-free mTORC1 is still capable of phosphorylating S6K (*73*), and a compensatory increase of FLCN and other Ragulator components has been previously observed in Ragulator-deficient cells, which—similar to our hepatocyte subpopulations—retained pS6 (*74*). This compensation may be caused by nuclear TFEB/TFE3, which binds to CLEAR motifs present in promoters of mTORC1-related genes, such as Rheb, Tsc1/2, Rrags, or Lamtor1-5 (*36*). Third, the TFEB/TFE3 loss-of-function models used here are chronic genetic models and therefore cannot fully exclude compensatory rewiring or engagement of alternative mTORC1-activating pathways. Acute TFEB/TFE3 loss- and gain-of-function experiments would be required to define the immediate causal contribution of MiT/TFE factors to pS6 signaling in this context. Fourth, our data on hyperactive mTORC1 rely primarily on *Tsc1* deficiency, which is an important limitation. Although Tsc1-LKO provides a robust genetic model of constitutive mTORC1 activation, *Tsc1* loss can also affect additional signaling and metabolic pathways and has been associated with liver injury, inflammation, and tumorigenesis in chronic liver models (*19*, *75*, *76*). Thus, the BA phenotype in this model should not be interpreted as a pure consequence of physiological mTORC1 activation. This limitation is illustrated by RagA-GTP mice, in which constitutive mTORC1 activation is not accompanied by altered DNL gene expression, detectable TFEB pathway activation, liver injury, or cancer despite partially overlapping effects on ketogenesis and PPAR signaling (*77*). Likewise, the deletion of *Tsc1* by Alb-Cre and AAV-Cre has divergent effects on VLDL secretion, FAO, and glucose sensitivity (*19*, *44*, *78*), which occur independently of FOXO1 activation. Hence, Tsc1-LKO mice may not be an ideal model for studying interventions that require intact upstream TSC regulation, including dietary PR or CRISPR-Cas9–mediated *Cyp8b1* knockdown. Future studies using TSC2-5A mutant mice may help dissect hyperactive mTORC1 signaling while preserving TSC-dependent upstream regulation (*46*, *79*). Along these lines, the deletion of *Tsc1* or *Flcn* by Alb-Cre results in severe liver damage and progenitor/cholangiocyte activation (*75*, *76*, *80*) as compared to AAV-Cre (*18*, *19*), which is in concurrence with PI3K/AKT/mTOR being a key regulator of cholangiocyte-to-hepatocyte plasticity in human MASLD (*81*, *82*). 12-OH BAs were elevated in both models, but we did not clarify whether 12-OH BAs are mediators of liver injury and tumorigenesis (*12*–*14*) in the context of Alb-Cre deletion (*75*, *76*, *80*). This phenotype contrasts that of *Lamtor2* and *Raptor* KO (Fig. 1S and fig. S5E) (*83*), with compensatory changes occurring by Alb-Cre deletion (Figs. 1E and *5J*) (*21*, *78*, *84*). Fourth, as some of our findings relate to regulation of *Cyp2c70*, a mouse specific gene, caution is advised in extrapolating our findings to humans. Whole body and liver cholesterol biosynthesis (16-fold and 4-fold higher in mice) as well as regulation of BA synthesis by dietary cholesterol differ tremendously between mice/rats and hamsters/humans (*85*, *86*). Similarly, rodent BAs, particularly MCAs, exert FXR antagonist effects, whereas CDCA, the predominant BA in humans, is a naturally occurring FXR agonist. Still, AAV-Lamtor2/Rptor may offer an opportunity to study suppressed mTORC1 signaling in progressive MASLD/MASH, as these mice show hepatocyte-to-cholangiocyte plasticity, shuttle cholesterol toward mitochondria, with alternative BA synthesis that results in a humanized BA pool, enriched with CDCA (*8*, *14*).

## MATERIALS AND METHODS

### Human samples

Human liver tissue and sera from patients were collected according to a published protocol after written informed consent was obtained from each participant and following approval by the Ethics Committee of the Medical University of Graz, Austria (EK 31-177 ex 18/19), in accordance with the Declaration of Helsinki. Blood was collected from 83 patients with liver disease with clinical and radiological signs and/or biopsy-proven nonalcoholic fatty liver disease (NAFLD) and/or nonalcoholic steatohepatitis (NASH). Patients with a Child-Pugh score > 11, alcohol abstinence of <2 weeks, clinical signs of active infection, Abx within 7 days before enrollment, gastrointestinal bleeding within the past 2 weeks, use of immunomodulatory agents within 1 month, renal insufficiency, renal failure, creatinine >1.5× the upper limit of normal, hepatic encephalopathy II to IV, pancreatitis, other organ failure, hepatic or extrahepatic malignancy, and pregnancy were excluded.

For the human high- versus low-protein diet experiment, human serum and liver samples from the LEMBAS trial were used. The trial was conducted at the Department of Clinical Nutrition of the German Institute of Human Nutrition Potsdam-Rehbrücke (Nuthetal, Germany), approved by the Ethics Committee of the Charité University Medicine in Berlin (application no. EA4/006/15), conducted in accordance with the Declaration of Helsinki, and registered at www.drks.de (DRKS00009509). Written informed consent was obtained from all participants before inclusion in the study. Details can be found elsewhere (*47*). In brief, 19 participants with morbid obesity who were undergoing bariatric surgery were divided into two groups on a hypocaloric diet. The HP group consumed 30% of energy from protein, 25 to 30% from fat, and 35 to 45% from carbohydrates (*n* = 9), whereas the LP group consumed 10% of energy from protein, 25 to 35% from fat, and 55 to 65% from carbohydrates (*n* = 10). This diet was maintained for 3 weeks before surgery at Vivantes Hospital Spandau (Berlin, Germany).

### Animal studies

All the experiments were approved by the European Directive 2010/63/EU and approved by the Austrian Federal Ministry of Education, Science and Research (Vienna, Austria; BMWFW- 66.010/0124-V/3b/2018 and BMWFW-66.010/0129-WF/V/3b/2016) and were in conformity with the Austrian Animal Welfare Act. Mice were bred and maintained under specific pathogen–free conditions. Cages were supplemented with nesting material and gnawing sticks and were changed once weekly. Health status of mice was monitored according to FELASA (Federation of European Laboratory Animal Science Associations) guidelines three times per year using the dirty bedding sentinel program. All animals were housed with a 12-hour light-dark cycle in humidity and temperature-controlled conditions and permitted ad libitum consumption of water and a standard mouse diet (Ssniff), except for special diets or periods of fasting where specified. For all studies, age- and sex-matched littermates were used as controls. Genotype, sex, age, and number of mice are indicated for each experiment in the appropriate figure legends.

*Lamtor2*^flox/flox^ (*87*), *Raptor*^flox/flox^ (*88*), and *Tsc1*^flox/flox^ (*89*) (the Jackson Laboratory, Stock: Tsc1tm1Djk/J; JAX strain number: 005680) mice were generated as described elsewhere. The animals were crossbred with Albumin-Cre mice (*90*) [the Jackson Laboratory, stock: B6.Cg-Speer6-ps1Tg(Alb-cre)21Mgn/J; JAX strain number: 003574] to obtain hepatoblast-specific *Lamtor2*, *Raptor*, or *Tsc1* KO mice (Ltor2-LKO, Rptor-LKO, and Tsc1-LKO). The Albumin-Cre allele was kept hemizygous. To detect and lineage trace LAMTOR2-deficient cells, we crossed our mice, as indicated in the respective figure legends, with the R26RtdTomatoLSL mouse [Ai9 or Ai9(RCL-tdT) (*91*); the Jackson Laboratory, stock: B6.Cg-Gt(ROSA)26Sortm9(CAG-tdTomato)Hze/J; JAX strain number: 007909], which is a Cre reporter tool strain. These mice have a STOP cassette flanked by loxP sites, preventing the transcription of the red fluorescent protein variant tdTomato located in the ubiquitously expressed ROSA26 locus under the control of the strong CAG promoter. Ai9 mice express robust tdTomato fluorescence after Cre-mediated recombination but exhibit only traces of tdTomato expression before exposure to Cre recombinase.

To induce acute gene KO in adult differentiated hepatocytes of *Lamtor2*^flox/flox^, *Raptor*^flox/flox^, *Tsc1*^flox/flox^, and tdTomato reporter mice, Cre-mediated recombination was induced with AAV.TBG.PI.Cre.rBG (a gift from J. M. Wilson; Addgene viral prep #107787-AAV8; http://n2t.net/addgene:107787; RRID:Addgene_107787). In control animals, either mice with WT alleles were injected with the same virus expressing Cre recombinase or floxed mice were injected with a mock virus lacking Cre recombinase, pAAV.TBG.PI.Null.bGH (a gift from J. M. Wilson, Addgene viral prep #105536-AAV8; http://n2t.net/addgene:105536; RRID:Addgene_105536). Viruses were administered by intraperitoneal injection of 2 × 10^11^ genome copies (GCs) per mouse diluted in 150 μl of sterile saline. Mice were euthanized 4 weeks after virus injection unless otherwise indicated in the respective figure legend.

To induce *Lamtor2* deficiency in *Tfeb*, *Tfe3* and DKO (BKO, 3KO, and DKO) mice, *Tfeb*^flox/flox^ (*92*) mice were crossed with whole-body *Tfe3* KO (*93*) mice to obtain *Tfeb*^flox/flox^ and *Tfe3* WT females and males that were either *Tfeb*^flox/flox^; *Tfe3*^+/Y^ or *Tfeb*^flox/flox^; *Tfe3*^-/Y^. At 8 weeks of age, these mice were injected intraperitoneally with 1 × 10^12^ GCs of an AAV expressing a single-guide RNA (sagRNA) and SaCas9 (VectorBuilder; pAAV[Exp]-U6 > mLamtor2[sagRNA#1]-TBG > SaCas9; sagRNA sequence (5′-3′): GCTAAGCCAAGCCAACACTGG; Vector ID: VB210525-1334ert), together with 2 × 10^11^ GCs of either an AAV expressing Cre recombinase (AAV.TBG.PI.Cre.rBG) or a mock virus without Cre recombinase (pAAV.TBG.PI.Null.bGH) to produce control mice (*Tfeb*^flox/flox^; *Tfe3*^+/Y^ with Null), BKO mice (*Tfeb*^flox/flox^; *Tfe3*^+/Y^ with Cre), 3KO mice (*Tfeb*^flox/flox^; *Tfe3*^−/Y^ with Null), and DKO mice (*Tfeb*^flox/flox^; *Tfe3*^−/Y^ with Cre), which are all *Lamtor2*-deficient. The mice were euthanized 5 weeks after the viral infection ad libitum.

To knockdown *Cyp8b1* using CRISPR-Cas9 in WT or Tsc1-LKO mice, 1 × 10^12^ GCs of an AAV expressing a sagRNA and SaCas9 (VectorBuilder; pAAV[Exp]-U6 > mCyp8b1[sagRNA#1]-HLP promoter>SaCas9; sagRNA sequence (5′-3′): GTAGGAGCCCTGCTCACGGTTG; Vector ID: VB221010-1356vkw) were injected intraperitoneally. Three days after the injection, the mice were fed a HSHCHF diet (Ssniff, E15723) for 10 weeks and then euthanized ad libitum.

For experiments involving fasting and refeeding, the mice were euthanized either immediately after an overnight fasting period or after overnight fasting followed by 2 or 6 hours of refeeding. For the MASLD experiment, mice were fed normal chow or HCHF (Ssniff, E15771) or HSHCHF (Ssniff, E15723) diets for 12 weeks and euthanized after a 1- to 2-hour fast (essentially ad libitum).

For dietary inactivation of mTORC1 by PR, mice were fed a protein-restricted diet (Ssniff, E15202) for 14 days and euthanized after a 1- to 2-hour fast (essentially ad libitum). For the BA experiments, mice received a diet enriched with 0.5% (w/w) CA for 1 week. Rapamycin-mediated inhibition of mTORC1 activity in Ltor2-LKO mice was achieved by a 0.02% (w/w) diet enriched with rapamycin for 1 week. Based on a daily dietary intake of 5 g, this would result in a daily dose of rapamycin (∼33 mg/kg) in an adult 30-g mouse. To investigate the effects of hyperactive mTORC1 signaling in Tsc1-LKO mice, mice were gavaged with rapamycin (30 mg/kg) or vehicle (olive oil) daily for 4 days. To rescue the BA phenotype in Ltor2/AAV-Cre mice, the animals were either treated with GW4064 and fed a diet containing 0.025% (w/w) GW4064 (treatment with this potent synthetic FXR agonist aimed to inhibit BA biosynthesis) or treated with an antibiotic cocktail [neomycin (100 μg/ml), ampicillin (100 μg/ml), ceftazidime (100 μg/ml), streptomycin (50 μg/ml), vancomycin (50 μg/ml), bacitracin (1 mg/ml), and metronidazole (10 μg/ml)] mixed into the drinking water. The antibiotic cocktail was given for 2 weeks, starting 2 weeks after virus injection (treatment aimed to inhibit microbiota-dependent BA deconjugation). Based on a daily food intake of 5 g, this would result in a daily dose of GW4064 (∼40 mg/kg) in an adult 30-g mouse.

After completion of the animal protocols, mice were anesthetized with isoflurane and euthanized by decapitation, and the blood was collected from the trunks. Organs were dissected, weighed, and either snap-frozen in liquid nitrogen or fixed in 4% paraformaldehyde for at least overnight and then embedded for histological workup. Frozen samples were stored at −80°C until further analysis and shipped on dry ice if needed.

For the experiments involving *Flcn* and *Tfe3* mice, mouse liver samples from the laboratory of Z. Arany were used. Male mice were fed normal chow (LabDiet, 5010) until they reached adulthood. Mice were injected with 1.5 × 10^11^ GCs per mouse of AAV8-TBG-GFP or AAV8-TBG-Cre (Penn Vector Core, AV-8-PV0146 and AV-8-PV1091) to produce control, FKO, *Tfe3* KO, and DKO mice. One week later, the mice were fed a normal chow/normal water or a fructose, palmitate, and cholesterol diet/sugar water regimen (Teklad, TD160785) for 16 weeks. Then, after removing their food (but not their normal or sugar water) for 4 to 6 hours, the mice were euthanized (*18*).

For the mRNA expression analysis during the neonatal period, mouse liver samples from the laboratory of Dr. Mathias Hornef were used. C57BL/6 J mice aged 0, 1, 4, 7, 11, 14, 21, 28, 63, and 105 days were euthanized ad libitum (*31*).

For the APAP-induced liver regeneration experiment, mouse liver samples from the laboratory of N. Henderson were used. Mice fasted for 12 hours before intraperitoneal injection of APAP [300 mg/kg (male) or 350 mg/kg (female)] dissolved in sterile phosphate-buffered saline (PBS) and euthanized after 36, 48 and 96 hours (*29*).

For the control versus TFEB overexpression experiment, mouse liver samples from the laboratory of A. Ballabio were used. Tfeb-3xFlagfs/fs mice were injected with a control or a Cre-expressing AAV (AAV8-TBG-Ctrl versus -Cre) and euthanized 4 weeks after injection (*48*).

### Intraperitoneal GTT

Mice were fasted overnight. The mice were then weighed, and tail blood was collected to measure fasting glucose using a glucometer. Each mouse received an injection of 20% d-glucose (10 μl per gram of body weight; corresponding to a final dose of 2 g/kg). Blood glucose levels were then measured at serial time points as indicated. To compare glucose excursion across individual animals, the area of the curve was calculated for each mouse.

### Isolation of primary hepatocytes

Sterile solutions and equipment were prepared. The solutions were prewarmed to 42°C in a water bath. Mice were anesthetized, and the abdomen was surgically opened through a vertical incision. The livers were perfused via the portal vein with prewarmed Krebs-Henseleit buffer II (115 mM NaCl, 25 mM NaHCO_3_, 5.9 mM KCl, 1.18 mM MgCl_2_, 1.23 mM NaH_2_PO_4_, 6 mM glucose; KHB II) for 5 to 10 min at a flow rate of 5 ml/min, followed by perfusion with KHB II containing collagenase type II (0.2 mg/ml; Worthington), 2% bovine serum albumin, and 0.1 mM CaCl_2_ for 10 min. The liver (without the gallbladder) was thereafter dissected, placed in collagenase solution, and minced, and the cell suspension was passed through a gauze, followed by washing with ice-cold KHB I (115 mM NaCl, 25 mM NaHCO_3_, 5.9 mM KCl, 1.18 mM MgCl_2_, 1.23 mM NaH_2_PO_4_, 1.2 mM Na_2_SO_4_, 1.25 mM CaCl_2_, and 6 mM glucose) and filtration through a 70-μm cell strainer. PMHs were separated from NPCs by centrifugation at 50 g for 3 min at 4°C. The supernatant containing the NPC fraction was centrifuged at 900 g for 5 min at 4°C. PMH and NPC were both cultivated in Dulbecco’s modified Eagle’s medium (DMEM) (Gibco, 41965) containing 10% fetal bovine serum (FBS) (Gibco, 10500064) and 1% penicillin/streptomycin (Gibco, 15140122). To induce gene KO in vitro, cells isolated from floxed animals were treated with either a control adenovirus or a Cre-expressing adenovirus (Ad5CMVeGFP versus Ad5CMVCre-eGFP).

### Cell culture

HepG2, Huh7, and HT29 cells were cultivated in DMEM (Gibco, 41965) containing 10% FBS (Gibco, 10500064) and 1% penicillin/streptomycin (Gibco, 15140122). Cells were treated with CDCA (100 μM), GW4064 (5 μM), and/or Torin1 (300 nM) for 16 to 18 hours as indicated.

### Serum biochemistry measurements

An automated biochemical analyzer (Hitachi 917) was used to determine mouse serum levels of ALT, AST, AP, TAGs, and cholesterol. Crystal Chem’s Ultra Sensitive Mouse Insulin ELISA Kit (Crystal Chem, 90080) was used to quantify mouse insulin levels in serum. Blood glucose levels were monitored with a Wellion CALLA Classic blood glucose meter.

### RNA isolation

RNA was extracted from frozen tissue samples using an appropriate amount of Trizol reagent (Invitrogen, 15596026) according to the manufacturer’s instructions. Liver samples were homogenized in a MagNA lyser at 6500 rpm twice for 20 s before extraction. The concentration and quality (absorbance ratios 260/280 and 230/280) of RNA were measured spectrophotometrically (NanoDrop 2000).

### cDNA production and RT-qPCR

The isolated RNA was reverse transcribed using the SuperScript II cDNA synthesis enzyme (Invitrogen, 18064022) with RNAse inhibitor (Applied Biosystems, N8080119) according to the manufacturer’s instructions. cDNA was diluted 1:20. For reverse transcription quantitative polymerase chain reaction (RT-qPCR), both forward and reverse primers were used at a concentration of 500 nM each, and Luna Universal qPCR Master Mix (New England Biolabs, M3003) was used on a LightCycler 480 to amplify the cDNA in a standard two-step PCR protocol. Specificity of the amplification of the qPCR products was verified by analysis of the melting curves. Relative changes were calculated using a standard curve, and gene expression was normalized to the geometric mean of at least two housekeeping genes for each sample per experiment (36b4, Uxt, and Hprt). For each gene, the resultant values were then normalized to the average CT among the biological replicates of controls. All primers were ordered from Eurofins Genomics in high purity salt-free (HPSF) purity. The primers used are listed in table S1.

### RNA sequencing

RNA sequencing on samples from human HepG2 cell line and mouse liver tissue was performed at the Next Generation Sequencing Core facility at the Medical University of Innsbruck on an Ion Proton device (Thermo Fisher Scientific) and using a QuantSeq 3′ mRNA library preparation kit (Lexogen) according to the manufacturer’s instructions. Trimmomatic was used to cut single-end sequencing reads at a length of 200 base pairs and to remove adaptor sequences and sequencing reads with minor quality as assessed by FastQC. The splice-aware aligner STAR (*94*) (v 2.5.3a, v 2.7.1a) was used to map reads to the UCSC human reference genome (hg38) (on average 10.4 million reads per sample could be uniquely mapped) and mouse reference genome (mm10) (on average, 5.3 million reads per sample could be uniquely mapped), respectively. HTSeq was used to assess raw read counts according to National Center for Biotechnology Information gene annotation (refGene). The R package DESeq2 (*95*) was used for normalization and to identify differentially expressed genes based on a negative binomial distribution. *P* values were adjusted for multiple testing based on the false discovery rate (FDR) according to the Benjamini-Hochberg method. Unless otherwise specified, genes which were more than twofold changed at a FDR < 0.1 and with mean of normalized counts > 10 were considered significantly differentially expressed. Heatmap visualization and hierarchical clustering was performed using Genesis (v. 1.8.1). The gene set enrichment analysis (*96*) (desktop version 4.2.3) was used to analyze whether hallmark gene sets (MSigDB) were significantly enriched based on preranked gene lists according to log_2_ fold changes.

### snRNA-seq including isolation of nuclei from mouse liver

The snRNA-seq was performed using a previously published workflow (*97*). Briefly, nuclei were isolated from flash-frozen livers using a modified DroNc-seq protocol published elsewhere (*98*). Tissue was minced to 1 mm^3^, homogenized in Nuclei EZ lysis buffer on ice, and filtered through a 40-μm cell strainer. It was then centrifuged at 500*g* for 5 min at 4°C, and the pellet was resuspended in PBS containing DAPI. The nuclear suspensions were loaded into a MoFlo Astrios EQ Cell Sorter and sorted into a 1.5-ml tube.

10X-based libraries were obtained using the Chromium Single Cell V3.0 reagent kit according to the manufacturer’s protocol (10X Genomics). Nuclei suspensions containing ∼500 nuclei/μl were loaded into nine independent lanes. Libraries were sequenced on a NovaSeq 6000 (Illumina). Computational preprocessing followed the previously published workflow (*97*). In brief, for data analysis, we first used CellBender to distinguish cell-containing droplets from empty droplets. We then applied Scrublet and DoubletFinder to exclude potential doublets, and nuclei expressing both male and female signature genes were excluded from downstream analysis. The mouse genome assembly GRCm38.p5 was used for mapping. CCA (*99*) from the Seurat package was used for batch correcting, clustering, and signature gene identification. SingleR was used to annotate cell clusters.

### Protein isolation from whole tissues

Tissue was lysed using a sonicator in a protein lysis buffer [homemade; 20 mM Hepes (pH 7.6) at room temperature (RT), 150 mM NaCl, and 0.5 to 1% Triton X-100] containing protease and phosphatase inhibitors (Thermo Fisher Scientific, A32959) at 4°C. The mixture was spun at 10 min at maximum speed (∼14,000 rpm) at 4°C to pellet debris. The supernatant was then collected into a fresh tube, taking care to avoid any top lipid layer. If the lipid layer was disturbed, then the tubes were respun, and the lipid-free supernatant was collected again. Total protein concentration in the tissue lysate was measured with the Pierce BCA Protein Assay Kit (Thermo Fisher Scientific, 23225) according to the manufacturer’s instructions.

### Immunoblotting

The same amount (15 to 30 μg) of total protein per sample was mixed with 2-mercaptoethanol– and SDS-containing loading buffer (i.e., a homemade standard Laemmli Buffer), denatured at 95°C for 5 min, and loaded into 7.5 to 15% SDS–polyacrylamide gel electrophoresis (SDS-PAGE) gels (homemade; depending on the size of the protein of interest) along with prestained protein ladders (Bio-Rad, 1610374). We ran our protein gels at 100 V (i.e., ∼20 mA per gel) for around 1.5 hours or until the migration front reached the bottom. The proteins were then transferred to a nitrocellulose membrane (Bio-Rad, 1620115) using a wet tank transfer method. To ensure uniform protein loading, the membrane was stained with Ponceau S (Sigma-Aldrich, P7170) and total protein normalization was recorded. The membrane was then blocked for at least 30 min at RT in blocking buffer [5 to 10% (w/v) nonfat dry milk powder in TBST]. The primary antibody against the target protein was added to the blocking buffer at an appropriate dilution (mostly 1:1000), and the membrane was incubated overnight at 4°C with agitation. After incubation, the membranes were washed briefly with TBST. A horseradish peroxidase–conjugated anti-rabbit (Cell Signaling Technology, 7074) or anti-mouse (Cell Signaling Technology, 7076) immunoglobulin G secondary antibody diluted 1:3000 was applied under agitation for 1 hour at RT. The blot was then washed again with TBST and finally incubated with ECL Western Blotting Substrate (Bio-Rad, 1705060). Chemiluminescence was detected using the Bio Rad ChemiDoc Touch Imaging System. Phosphorylated proteins were normalized to the total amount of each protein; otherwise, Ponceau S staining served as a loading control. Phos-tag SDS-PAGE was performed according to the manufacturer’s instructions using a resolving gel solution containing 0.1 mM of MnCl_2_ and 50 μM of Phos-tag acrylamide (Wako, 304-93523). The following primary antibodies were used: anti-ACLY (Proteintech, 15421-1-AP), anti-ACSS2 (Cell Signaling Technology, 3658), anti-CHREBP (Novus Biologicals, NB400-135), anti-CTNNB1 (Cell Signaling Technology, 9587), anti-CYP27A1 (Abcam, ab126785), anti-CYP2C22/CYP2C70 [kind gift from E. T. Morgan (*100*)], anti-CYP7A1 (Merck Millipore, MABD42), anti-CYP7B1 (Abcam, ab138497), anti-CYP8B1 (Abcam, ab191910; Invitrogen, PA5-37088), anti-FASN (Cell Signaling Technology, 3180), anti-FLCN (Cell Signaling Technology, 3697), anti-FXR/NR1H4 (R&D Systems, PP-A9033A-00), anti-HNF4α (Cell Signaling Technology, 3113), anti-LAMTOR2 (Cell Signaling Technology, 8145), anti-LAMP1 (Abcam, ab24170), anti-LC3 (Novus Biologicals, NB100-2220), anti-mTOR (Cell Signaling Technology, 2983), anti-phospho AMPK Thr^172^ (Cell Signaling Technology, 2535), anti–phospho S6 Ribosomal Protein Ser^235/236^ (Cell Signaling Technology, 2211), anti–phospho S6 Ribosomal Protein Ser^240/244^ (Cell Signaling Technology, 5364), anti-RAGA (Cell Signaling Technology, 4357), anti-RAPTOR (Cell Signaling Technology, 2280), anti-S6 Ribosomal Protein (Cell Signaling Technology, 2217), anti-SOX9 (Merck Millipore, AB5535), anti-TFE3 (Cell Signaling Technology, 14779), anti-TSC1 (Cell Signaling Technology, 6935), anti-TSC2 (Cell Signaling Technology, 4308), anti-VINCULIN (Cell Signaling Technology, 13901), and anti-YAP (Cell Signaling Technology, 14074).

### Histology analysis

Tissues were fixed in 4% paraformaldehyde for at least 16 hours, then dehydrated, and subsequently embedded in paraffin. Formalin-fixed, paraffin-embedded liver samples were cut into 2- to 5-μm-thick sections and stained with hematoxylin and eosin (H&E) or periodic acid-Schiff stain according to standard procedures. For immunohistochemistry, the sections were deparaffinized and then boiled in antigen retrieval solution for 10 min. After cooling, sections were blocked for 1 hour and then incubated with primary antibody for 2 hours at RT and secondary antibody for 1 hour at RT. Samples were counterstained with Mayer’s hematoxylin to identify cell nuclei using standard histological procedures. For immunofluorescence, tissues were fixed in 4% paraformaldehyde for ∼1 hour at 4°C and then cryopreserved in sucrose solution overnight. Samples were then frozen in liquid nitrogen and processed according to standard immunofluorescence procedures, including permeabilization, blocking, and antibody incubation. Sections were stained with DAPI (4′,6-diamidino-2-phenylindole) to visualize cell nuclei. Confocal images of the samples were acquired using a Nikon A1R+ confocal microscope. Images were analyzed using Fiji ImageJ. The following primary antibodies were used for histological analyses: anti-CYP2C22/CYP2C70 [kind gift from E. T. Morgan (*100*)], anti-CYP7A1 (Merck Millipore, MABD42), anti-CYP8B1 (Abcam, ab191910; Invitrogen, PA5-37088), anti-HNF4α (Cell Signaling Technology, 3113), anti–glutamine synthetase (Abcam, ab49873), anti–Ki-67 (Cell Signaling Technology, 12202), anti-LAMTOR2 (Cell Signaling Technology, 8145), anti-PCK1/PEPC (Abcam, ab70358), anti–phospho S6 Ribosomal Protein Ser235/236 (Cell Signaling Technology, 4858), anti-RFP (Rockland, 600-401-379-RTU), anti-SOX9 (Merck Millipore, AB5535), anti-TFE3 (Cell Signaling Technology, 14779), and anti-TFEB (Bethyl Laboratories, A303-673A).

### SIM imaging

The SIM-setup used is composed of a 405-, 488-, 515-, 532-, and a 561-nm excitation laser introduced at the back focal plane inside the SIM-box with a multimodal optical fiber. For super-resolution, a CFI SR Apochromat TIRF 100× oil (NA 1.49) objective was mounted on a Nikon-Structured Illumination Microscopy (N-SIM, Nikon, Austria) System with standard wide field and SIM filter sets and equipped with two Andor iXon3 EMCCD cameras mounted to a Two Camera Imaging Adapter (Nikon Austria, Vienna, Austria). For calibration and reconstruction of SIM images, the Nikon software (NIS-Elements, Nikon, Austria) was used. Reconstruction was permanently performed with the same robust setting to avoid artefact generation and ensures reproducibility with a small loss of resolution of 10% compared to most sensitive and rigorous reconstruction settings. The colocalization between OPA1 (Abcam, ab42364) and cholesterol (Sigma-Aldrich, F9765) was determined with the Pearsons correlation coefficient using ImageJ and the plugin coloc2.

### Electron microscopy

Electron microscopy of liver samples was performed according to a previously published protocol (*101*). Briefly, liver tissue that had been previously dissected, flash-frozen in liquid nitrogen, and stored without cryoprotective agents was used for analysis. Frozen tissue samples were cut into ∼5 mm^3^ pieces and fixed at 37°C in a solution containing 2.5% (w/v) glutaraldehyde and 2% (w/v) paraformaldehyde for 3 hours. Samples were then postfixed in 2% (w/v) osmium tetroxide for 2 hours at RT. After dehydration (in graded series of ethanol), tissues were infiltrated in propylene oxide (Sigma-Aldrich, US) and TAAB embedding resin overnight, afterward in pure TAAB embedding resin (3 hours) (TAAB Laboratories Equipment Ltd., UK) transferred into embedding moulds, and polymerized (48 hours, 60°C). Ultrathin sections (70 and 300 nm) were cut with a UC7 Ultramicrotome (Leica Microsystems, Austria) and stained with platinum blue (EMS, USA) for 15 min and lead citrate for 5 min. Electron micrographs and electron tomograms were taken using a Tecnai G2 transmission electron microscope (Thermo Fisher Scientific, Netherlands) with a Gatan Ultrascan 1000 charge coupled device (CCD) camera (−20°C, acquisition software Digital Micrograph, Ametek Gatan, Germany and Serial EM). The acceleration voltage was 120 and 200 kV. Electron tomograms were generated by the use of the SERIAL EM programme (Mastronarde 2003), and the measured tilt angles covered a span of ±60° with tilt angle increments of 1° resulting in 121 projection images. A 3D reconstruction of a tilt series was done with FLARA (*102*) to align the collected images. Image segmentation was performed using AMIRA software.

### Scanning transmission electron microscopy

Further investigations were performed using a scanning electron microscope. Scanning transmission electron microscopy (STEM) imaging mode was applied on a field emission scanning electron microscope (ZEISS FE-SEM Sigma 500) at an acceleration voltage of 15 kV, in combination with the ATLAS system. High-resolution overview images of large liver tissue regions were acquired with this setup and used to identify areas of interest for subsequent detailed electron microscopy imaging.

### MALDI-MS-imaging

Matrix-assisted laser desorption/ionization–mass spectrometry imaging (MALDI-MSI) was performed as described (*103*). Briefly, liver tissues specimens from the same lobe were frozen and fixed in Tissue-Tek OCT compound. Then, two adjacent 5-μm-thick frozen liver sections were prepared using a Leica CM3050 S cryostat (Leica, Wetzlar, Germany). The sections were then thawed, mounted on IntelliSlides, and dried in a desiccator. The slides were sprayed with four layers of 2-mercaptobenzothiazole (5 mg/ml) in a 5:1 acetone/water solution using an HTX imaging sprayer (HTX Technologies LLC, Chapel Hill, NC, USA) at 30°C and 10-psi nitrogen. One section was then processed for MALDI imaging, and the other was stained with H&E. MALDI measurements were performed at the MS1 level using a TIMSTOF fleX (Bruker Daltonics, Bremen, Germany) in negative mode, without ion mobility separation, within a mass range of 100 to 800 mass/charge ratio (*m*/*z*). Internal calibration was performed using 2-mercaptobenzothiazole matrix peaks and taurocholate. The data were interpreted using SCiLS Lab MVS (version 2021c). Last, the MALDI image was overlaid with the H&E-stained image to visualize the spatial distribution of the TCA/TMCA and TDCA/TCDCA signals.

### Quantification of hepatic lipid species

Lipid extraction was carried out from mouse liver samples according to a modified version of the original extraction protocol published elsewhere (*104*). Methanol (1.5 ml) and MTBE (2.5 ml) were added to the samples (about 50 mg) in 12-ml glass tubes with teflon lined caps. Tissue was homogenized for 30 s using the Ultra-Turrax tissue homogenizer (IKA Works Inc., Wilmington, N.C., USA). After the addition of 2.5 ml of MTBE and shaking for 10 s, the mixture was incubated in an ice-cooled ultrasound bath for 10 min. After the addition of 1.25 ml of deionized water and 10 min of overhead shaking, the mixture was centrifuged for 10 minutes at 1350*g* at RT, and the upper phase was transferred to a fresh glass tube. The lower phase was re-extracted with 2 ml of the upper phase of MTBE/methanol/deionized water (10:3:2.5, v/v/v), and again the upper phase was collected, combined with the upper phase from the first extraction, evaporated in a vacuum centrifuge (Thermo Fisher Scientific, Waltham, MA, USA), dissolved in 1000 μl of chloroform/methanol (1:1, v/v), and stored at −80°C. Before analysis, an aliquot of 100 μl of the storage solvent was evaporated under a gentle stream of nitrogen, and the samples were reconstituted in the same volume of injection solvent consisting of isopropanol/chloroform/methanol (90:5:5, v/v/v).

Chromatographic separation was performed as previously described (*105*). Briefly, chromatographic separation was performed on a Waters (Waters, Milford, MA, USA) BEH C8 column (100 mm by 1 mm, 1.7 μm), thermostatted to 50°C in a Dionex Ultimate 3000 RS UHPLC system. Mobile phase A was deionized water containing 1 vol % of 1 M aqueous ammonium formate (final concentration of 10 mM) and 0.1 vol % of formic acid as additives. Mobile phase B was a mixture of acetonitrile/isopropanol 5:2 (v/v) with the same additives. Gradient elution started at 50% mobile phase B, rising to 100% B over 40 min; 100% B were held for 10 min and the column was re-equilibrated with 50% B for 8 min before the next injection. The flow rate was 150 μl/min, the samples were kept at 8°C, and the injection volume was 2 μl.

The Orbitrap Velos Pro hybrid mass spectrometer (Thermo Fisher Scientific Inc., Waltham, MA, USA) was operated in data-dependent acquisition. Every sample was measured once in positive polarity and once in negative using a HESI II ion source. Ion source parameters for positive polarity were as follows: source voltage of 4.5 kV, source temperature of 275°C, sheath gas of 25 arbitrary units, Aux gas of 9 arbitrary units, sweep gas of 0 arbitrary units, and capillary temperature of 300°C. Ion source parameters for negative polarity were as follows: source voltage of 3.8 kV, source temperature of 325°C, sheath gas of 30 arbitrary units, Aux gas of 10 arbitrary units, sweep gas of 0 arbitrary units, and capillary temperature of 300°C. Automatic gain control target value was set to 106 ions to enter the mass analyzer, with a maximum ion accumulation time of 500 ms. Full scan profile spectra from *m*/*z* 320 to 1050 for positive ion mode and from 350 to 1600 in negative ion mode were acquired in the Orbitrap mass analyzer at a resolution setting of 100 000 at *m*/*z* 400. For MS/MS experiments in positive and negative ion mode, the 10 most abundant ions (top 10) of the full scan spectrum were sequentially fragmented in the ion trap using He as collision gas (collision-induced dissociation, normalized collision energy of 50; isolation width of 1.5, activation *Q* of 0.2, and activation time of 10), and centroid product spectra at normal scan rate (33 kDa/s) were collected.

LC/MS data were processed using lipid data analyzer (*106*). Briefly, the algorithm identifies lipids with a 3D algorithm using the three dimensions *m*/*z*, retention time, and intensity to correctly integrate peaks while also taking into account the isotopic distribution. MS/MS spectra are considered for confirmation of structures by characteristic head group and fatty acyl fragments.

### FPLC serum fractionation for serum lipoprotein profiles

Pooled serum (200 μl) of each genotype was subjected to fast protein liquid chromatography (FPLC) on a Pharmacia P-500 FPLC system equipped with a Superose 6 column. Lipoproteins were eluted with 10 mM tris-HCl, 1 mM EDTA, 0.9% NaCl, and 0.02% NaN_3_ (pH 7.4). Fractions of 0.5 ml each were collected, and TAG and TC concentrations were determined enzymatically using colorimetric kits according to the manufacturer’s instructions (triglyceride FS, REF 1 5760 00 10 023 and cholesterol FS, REF 1 1300 99 10 023, both from DiaSys). To increase sensitivity, the reaction buffers were supplemented with the addition of sodium 3,5-dichloro-2-hydroxy-benzenesulfonate (3 and 6 mg/ml; Sigma-Aldrich, D4646) for the determination of TAGs and cholesterol, respectively.

### Extraction and LC-MS of BAs from serum, liver, and bile

The sample preparation and liquid chromatography–mass spectrometry (LC-MS) protocol have already been published in detail (*107*). Briefly, bile had to be diluted 1:1,000 with distilled water before taking 10 μl for further sample processing. Serum (10 μl) was used without prior dilution. For extraction of liver BAs, 50 to 100 mg of liver tissue were homogenized in 200 μl of sterile saline using a sonicator. Polar/hydrophilic and apolar/lipophilic metabolites were separated using Folch’s extraction procedure. To the homogenate, 3.5 ml of chloroform:methanol (2:1) was added and incubated for at least 1 hour at RT with constant shaking. A 500 μl of 0.9% normal saline was added to induce phase separation, which was aided by centrifugation at 2000 rpm for 15 min at RT. The apolar lower phase was kept for neutral lipid analysis. The upper polar phase was dried in a SpeedVac Concentrator and reconstituted in 1 ml of methanol. A 30 μl of this solution was used for BA measurement. Internal standards were then added to serum, liver, or bile aliquots followed by deproteinization with acetonitrile. Samples were centrifuged, the supernatant was removed, dried, and dissolved in running buffer. BAs were separated by HPLC using a C18 reversed-phase column before quantification by MS.

### Measurement of hepatic glycogen content

The liver tissue (10 to 20 mg) was homogenized in 500 μl of 2 M HCl using a sonicator and boiled at 95°C for at least 1 hour to breakdown glycogen into its glucose subunits by acid hydrolysis. After cooling to RT, the homogenates were neutralized with 500 μl of 2 M NaOH and centrifuged at maximum speed for 10 min in a standard benchtop centrifuge. The glucose concentration was determined with the D-Glucose HK Assay Kit (Megazyme, 700004298) using the microplate assay procedure according to the manufacturer’s instructions. Preliminary experiments have shown that the determination of free glucose in the liver by alkaline hydrolysis of the tissue with NaOH followed by neutralization to preserve glycogen is not necessary because the amount of free liver glucose is negligible under the experimental conditions we studied.

### Statistical analysis, figure creation, and data availability

No statistical methods were used to determine the sample size in advance. Mice were randomized to experimental groups. Investigators were not blinded during experiments and outcome assessment. Data collection was performed using Microsoft Excel 2016 and GraphPad Prism 8.0 was used to prepare the graphical plots and statistical analysis. The statistical tests used are indicated for each experiment in the corresponding figure legends. A *P* value of less than 0.05 was considered statistically significant. The figure legends indicate the statistical tests used. Data are presented as means ± SEM for biological replicates, unless otherwise stated.

All RNA-seq data supporting this study have been deposited in ArrayExpress. The snRNA-seq data from mouse liver cells generated by 10X sequencing are available under accession code E-MTAB-16330 at www.ebi.ac.uk/biostudies/ArrayExpress/studies/E-MTAB-16330. Bulk RNA-seq data from HepG2 cells are available under accession code E-MTAB-16150 at www.ebi.ac.uk/biostudies/ArrayExpress/studies/E-MTAB-16150.
