# Supplementary material for: mTORC1-TFEB/TFE3 signaling is associated with bile acid diversification during hepatic metabolic adaptation
Source: Sci Adv. 2026 Sep 25;12(39):eaee1905. doi: 10.1126/sciadv.aee1905 (PMC13618816; doi:10.1126/sciadv.aee1905)
Supplement: Supplementary file 1 [file sciadv.aee1905_sm.pdf]

Science Advances
AAAS

Supplementary Materials for

mTORC1-TFEB/TFE3 signaling is associated with bile acid diversification during hepatic metabolic adaptation

Alex Zaufel *et al.*

Corresponding author: Tarek Moustafa, tarek.moustafa@medunigraz.at

Sci. Adv. **12**, eaee1905 (2026)
DOI: 10.1126/sciadv.aee1905

This PDF file includes:

Figs. S1 to S12
Table S1

Fig. S1. Lamtor2 deletion alters liver architecture, glycogen storage, and nutrient-responsive mTORC1 signaling.

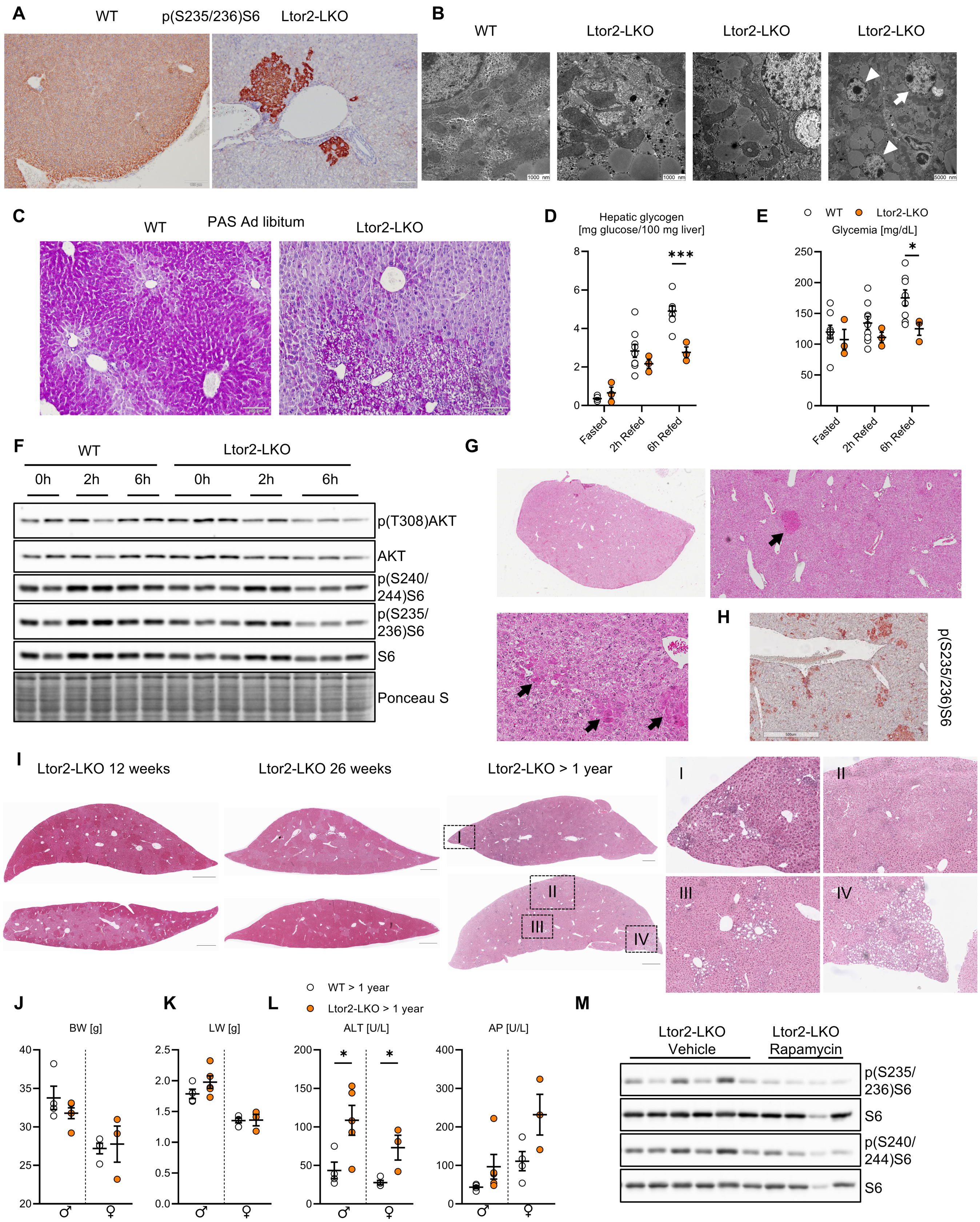

Fig. S1. Lamtor2 deletion alters liver architecture, glycogen storage, and nutrient-responsive mTORC1 signaling. (**A**) Representative immunohistochemical images of liver sections from wild-type (WT) and Ltor2-LKO mice refed for 2 hours and stained for serine 235/236 phosphorylated-S6. Scale bars, 100 µm. (**B**) Electron micrographs of the different hepatocyte populations found in WT and Ltor2-LKO mice. Arrowheads denote small hepatocellular nuclei. Arrow indicates a normal hepatocellular nucleus. (**C**) Representative periodic acid-Schiff (PAS) staining of liver sections from WT and Ltor2-LKO mice. Scale bar, 50 µm. (**D** to **F**) Female 10- to 16-week-old WT and Ltor2-LKO mice were euthanized after overnight fasting or after two or six hours of refeeding (n = 3-9). (D) Quantification of liver glycogen content and (E) serum glucose levels. (F) Phosphorylation of AKT at threonine 308 and the mTORC1 target S6 at serine 235/236 and 240/244 in livers. (**G** and **H**) Representative images of (G) liver H&E staining and (H) immunohistochemistry for serine 235/236 phosphorylated-S6 from 4-week-old Ltor2-LKO mice. Arrows indicate foci/cluster of pS6-positive hepatocytes on H&E. Scale bars, 500 µm. (**I**) Representative images of liver H&E staining from Ltor2-LKO mice at the indicated ages. Zoom II shows the remains of a cluster of small hepatocytes. Zoom I, III and IV show multifocal bile duct hyperplasia. Scale bars, 1 mm. (**J** to **L**) Male and female Ltor2-LKO mice and their WT littermates were fed a normal chow diet and euthanized at 1 to 1.5 years of age (n = 3-5). (J) Body weight (BW) in grams. (K) Liver weight (LW) in grams. (L) Serum examination of alanine aminotransferase (ALT) and alkaline phosphatase (AP) levels. (**M**) Phosphorylation of mTORC1 target S6 at serine 235/236 and 240/244 in livers of aged WT and Ltor2-LKO mice (> 1 year old) fed either normal chow or a diet containing 0.02% rapamycin for 1 week before euthanasia. $^*P < 0.05$, $^{**}P < 0.01$, $^{***}P < 0.001$, $^{****}P < 0.0001$. Statistical significance was calculated using a two-tailed unpaired Student's t test (J-L) or a two-way ANOVA followed by Holm-Šídák's multiple comparisons post hoc testing (D-E). Data are depicted as mean ± SEM.

Fig. S2. Marker-gene analysis annotates liver cell populations in the snRNA-seq dataset.

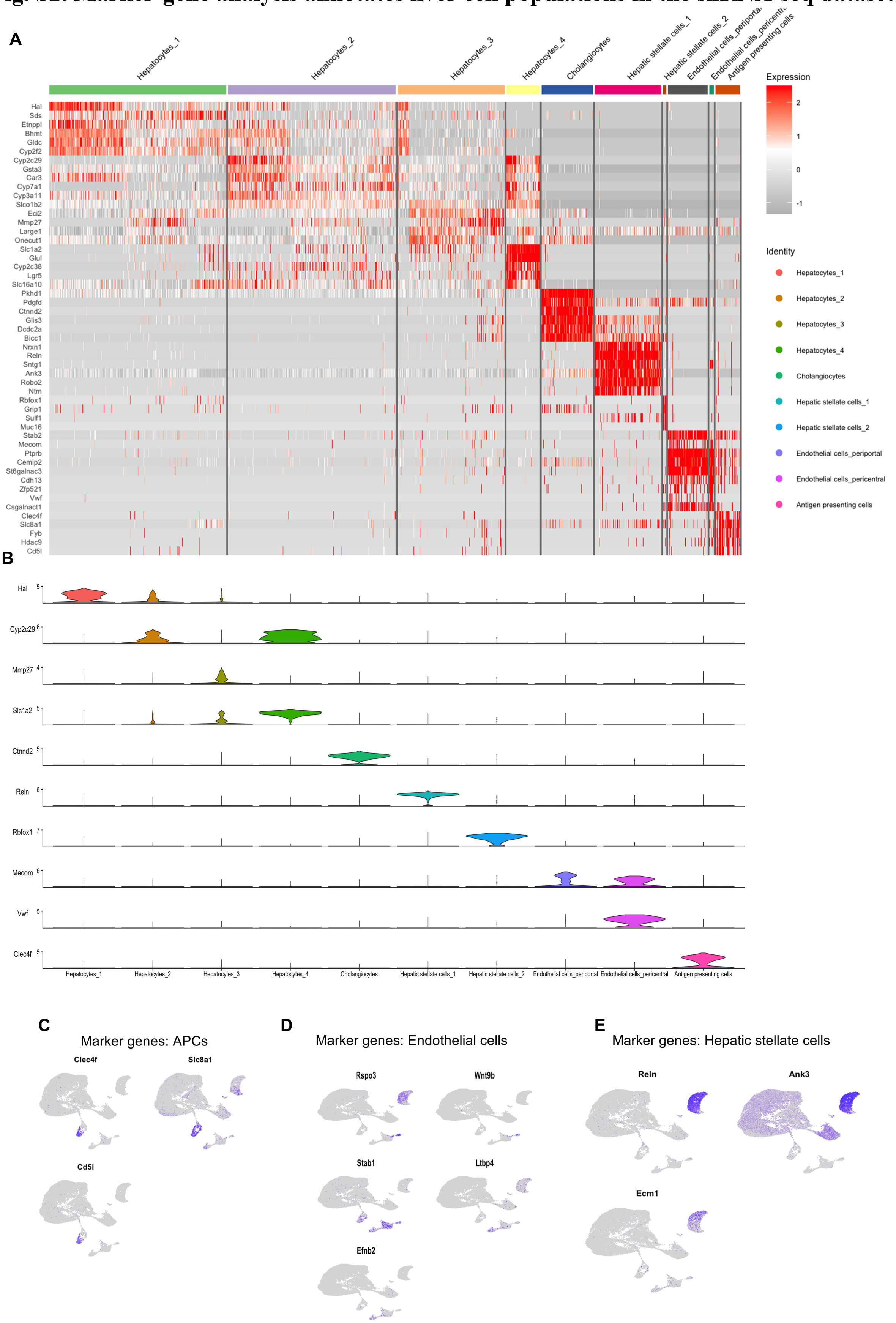

Fig. S2. Marker-gene analysis annotates liver cell populations in the snRNA-seq dataset. (**A**) Heat map of signature genes for each population of liver cells. (**B**) Violin plot of Hal, Cyp2c29, Mpm27, Slc1a2, Ctnnd2, Reln, Rbfox1, Mecom, Vwf and Clec4f. (**C** to **E**) Feature plots of marker genes for (C) antigen presenting cells (APCs), (D) endothelial cells and (E) hepatic stellate cells.

Fig. S3. Hepatocyte subclustering reveals genotype-associated transcriptional programs.

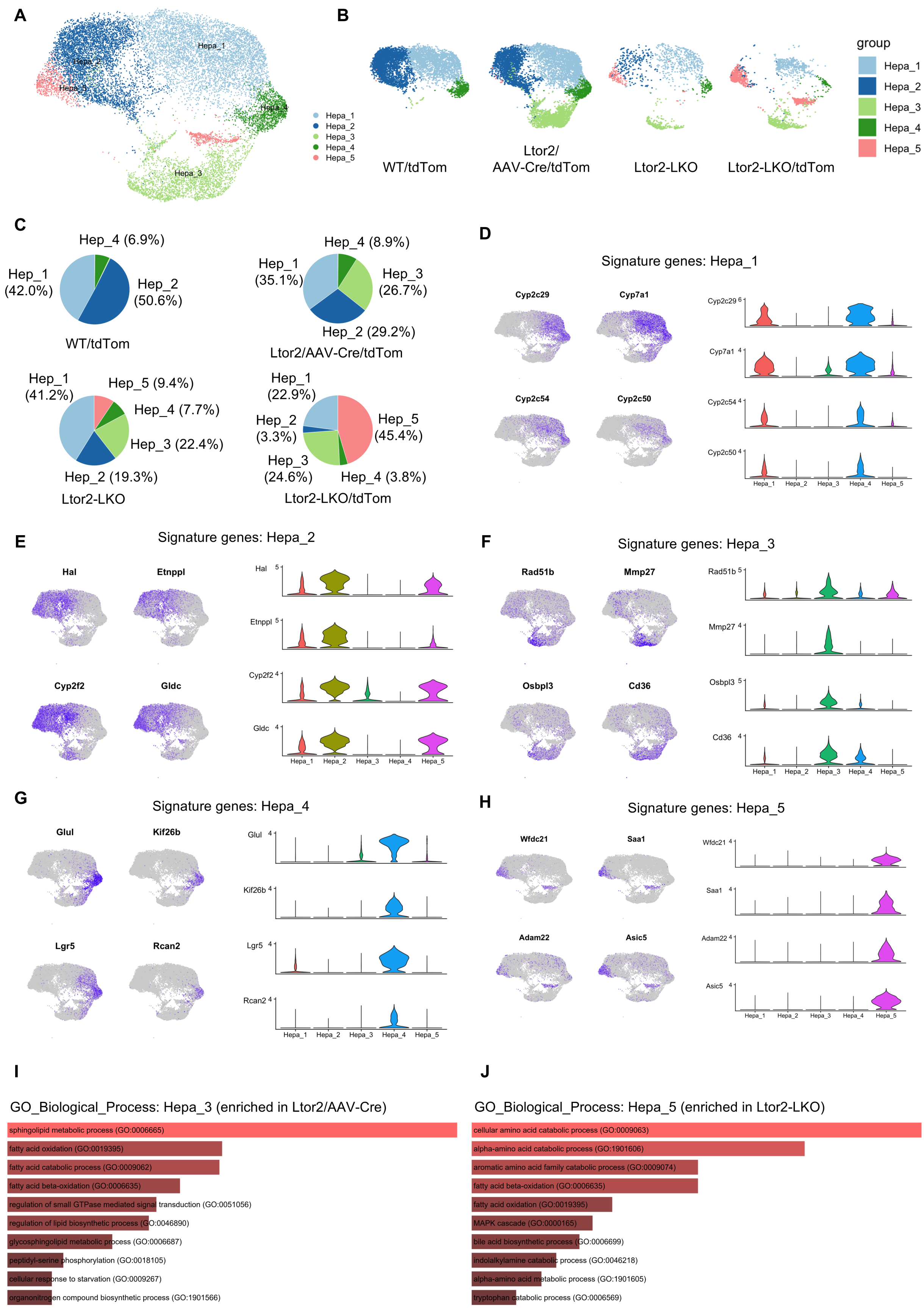

Fig. S3. Hepatocyte subclustering reveals genotype-associated transcriptional programs. (**A**) Unsupervised subclustering of 15,538 hepatocyte nuclei. Five subpopulations of murine hepatocytes pooled from four genotypes are shown. (**B**) Subcluster compositions in the four genotypes as indicated. (**C**) Pie charts show the relative contribution of each population to the total number of nuclei found in each of the four genotypes. Hepatocellular subpopulations are labelled (Hep_1-5), and the percentage of nuclei to the total number of nuclei is indicated in the parentheses. (**D** to **H**) Feature and violin plots of four signature genes primarily enriched in different hepatocyte subclusters. (**I** and **J**) Bar plots of the *P* value for GO term enrichment (Biological Process) for differentially expressed genes enriched in hepatocyte subclusters 3 and 5 (Hepa_3 and Hepa_5).

Fig. S4. Lamtor2 deficiency alters hepatocyte identity, zonation markers, and developmental gene programs.

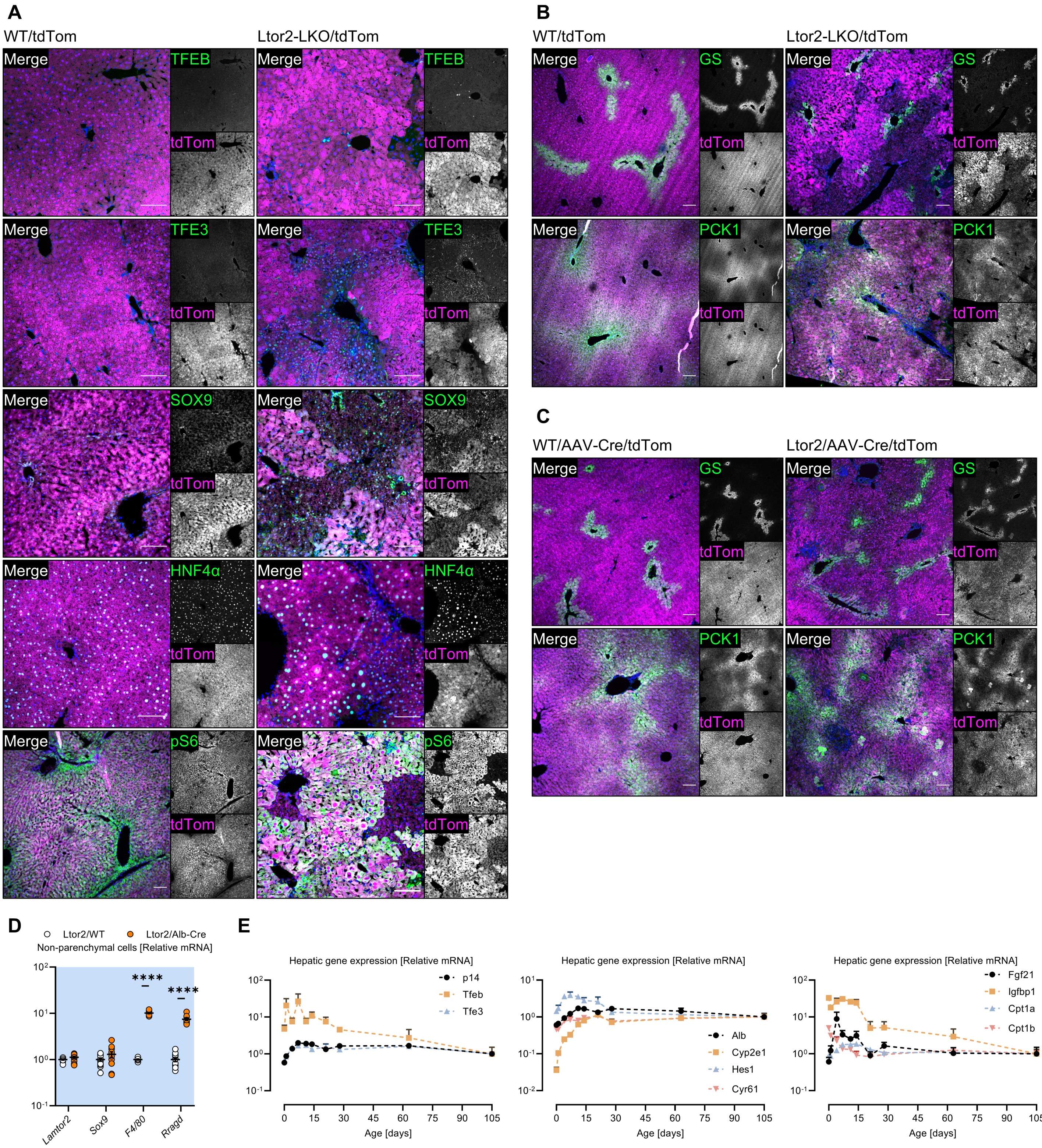

Fig. S4. Lamtor2 deficiency alters hepatocyte identity, zonation markers, and developmental gene programs. (**A**) tdTom/TFEB, tdTom/TFE3, tdTom/SOX9 (biliary marker), tdTom/HNF4α (hepatocellular marker) and tdTom/pS6 immunofluorescent confocal images in control and Ltor2-LKO mice. Images are representative of at least three male and female mice per condition. Scale bars, 100 µm. (**B**) tdTom/GS and tdTom/PCK1 immunofluorescent confocal images in Ltor2-LKO and control mice. Images are representative of at least three male as well as female mice per condition. Scale bars, 100 µm. (**C**) tdTom/GS and tdTom/PCK1 immunofluorescent confocal images in Ltor2/AAV-Cre or control mice four weeks after virus injection. Images are representative of at least three male as well as female mice per condition. Scale bars, 100 µm. (**D**) mRNA expression of *Lamtor2*, *Sox9*, *F4*/80 and *Rragd* in NPCs isolated from livers of Ltor2-LKO and control mice (n = 1 2 from at least two different mice per condition). (**E**) mRNA expression of *Lamtor2* (*p14*), *Tfeb* and *Tfe3*, hepatobiliary and mesenchymal marker genes, secretory proteins and fatty acid oxidizing enzymes during neonatal period. PND1-PND105 (n = 3-5 per time point). *$P < 0.05$, **$P < 0.01$, ***$P < 0.001$, ****$P < 0.0001$. Statistical significance was calculated using a two-tailed unpaired Student's t test (B). Data are depicted as mean ± SEM.

Fig. S5. Adult hepatocyte Raptor deletion recapitulates metabolic remodeling and pS6-positive hepatocyte foci.

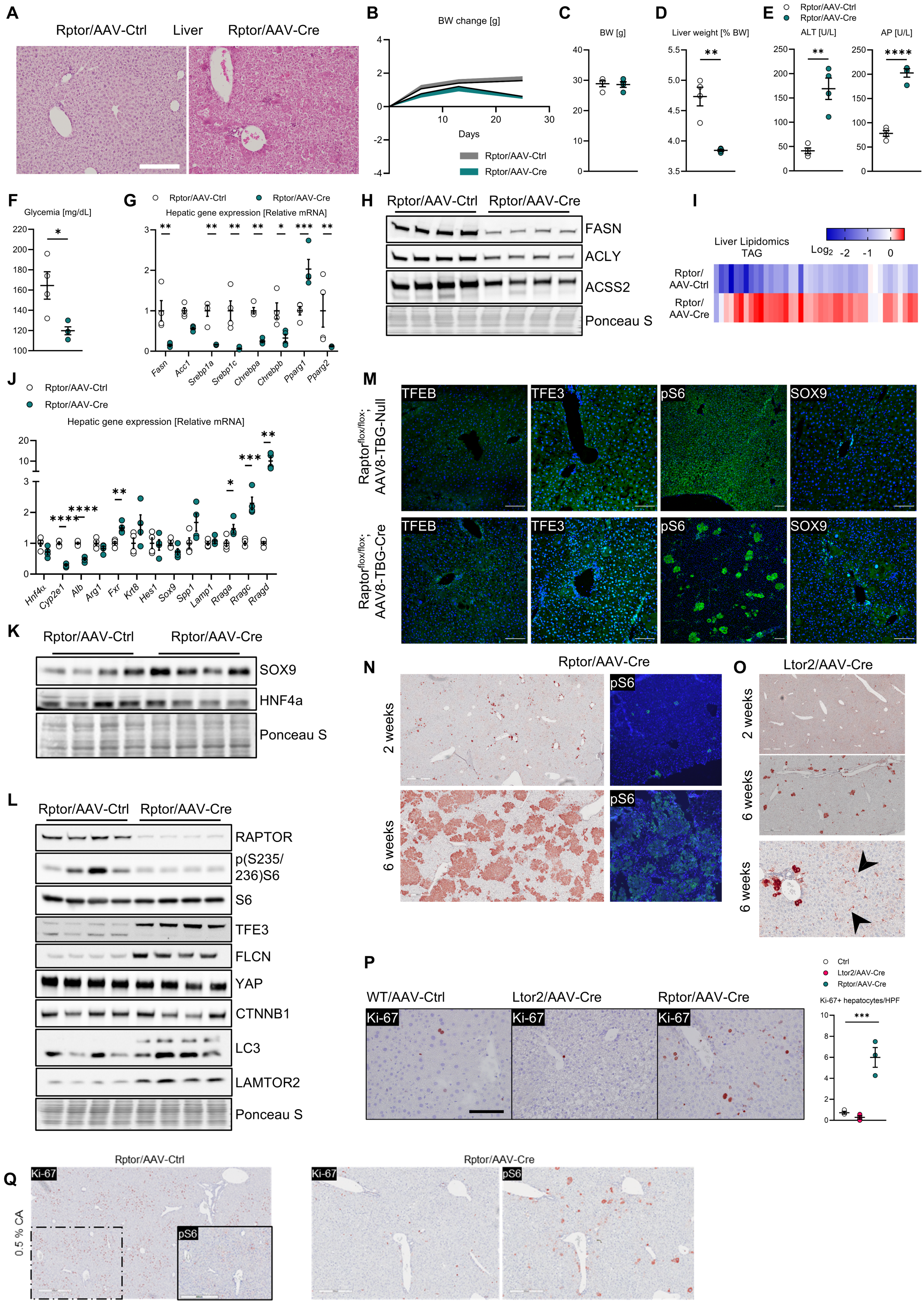

Fig. S5. Adult hepatocyte Raptor deletion recapitulates metabolic remodeling and pS6-positive hepatocyte foci. (**A** to **M**) Male Rptor/AAV-Ctrl and Rptor/AAV-Cre mice were fed normal chow and euthanized four weeks after virus injection (n = 4). (A) Representative images of liver H&E staining from Rptor/AAV-Ctrl and Rptor/AAV-Cre mice. Scale bars, 100 µm. (B) Change of body weights in grams after virus injection over time. (C) Body weight (BW) in grams. (D) Relative liver weight (LW) expressed as percent of total BW. (E) Serum examination of alanine aminotransferase (ALT) and alkaline phosphatase (AP) levels. (F) Serum glucose levels. (G) mRNA expression of hepatic genes involved in lipogenesis. (H) Western blot analysis of protein expression of lipogenic proteins from liver extracts. (I) Heatmap shows the mean abundance of hepatic triacylglyceride (TAG) species measured by LC-MS (n = 3). (J) mRNA expression of hepatobiliary marker and lysosomal marker genes. (K) Western blot analysis of protein expression of SOX9 and HNF4α from bulk liver extracts. (L) Western blot analysis of protein expression of lysosome-associated proteins from bulk liver extracts. (M) Confocal imaging analysis of immunofluorescence of liver sections stained for TFEB, TFE3, pS6 and SOX9. Images are representative of at least three mice per genotype. Scale bars, 100 µm. (**N**) Representative immunohistochemical and immunofluorescent images of liver sections from Rptor/AAV-Cre mice 2 and 6 weeks after virus injection stained for pS6. Scale bars, 500 µm. (**O**) Representative immunohistochemical images of liver sections from Ltor2/AAV-Cre mice 2 and 6 weeks after virus injection stained for pS6. Scale bars, 500 µm. (**P**) Immunohistochemical analysis of Ki-67 in livers of Ltor2/AAV-Cre mice and Rptor/AAV-Cre mice compared with controls. Scale bar, 100 µm. Hepatocellular Ki-67 was quantified. Images are representative of at least three mice per genotype. For quantification of Ki-67, Ki-67-positive hepatocyte nuclei from at least ten high-power fields (HPFs) from three mice per group were manually counted. (**Q**) Representative immunohistochemical images of liver sections from Rptor/AAV-Ctrl and Rptor/AAV-Cre fed a 0.5% CA-enriched diet stained for Ki-67 and pS6. Scale bars, 300 µm. *$P < 0.05$, **$P < 0.01$, ***$P < 0.001$, ****$P < 0.0001$. Statistical significance was calculated using a two-tailed unpaired Student's t test (C-G,J) or a one-way ANOVA followed by Holm-Šídák's multiple comparisons post hoc testing (P). Data are depicted as mean ± SEM.

Fig. S6. pS6-positive hepatocytes emerge at the regenerating interface after acetaminophen injury.

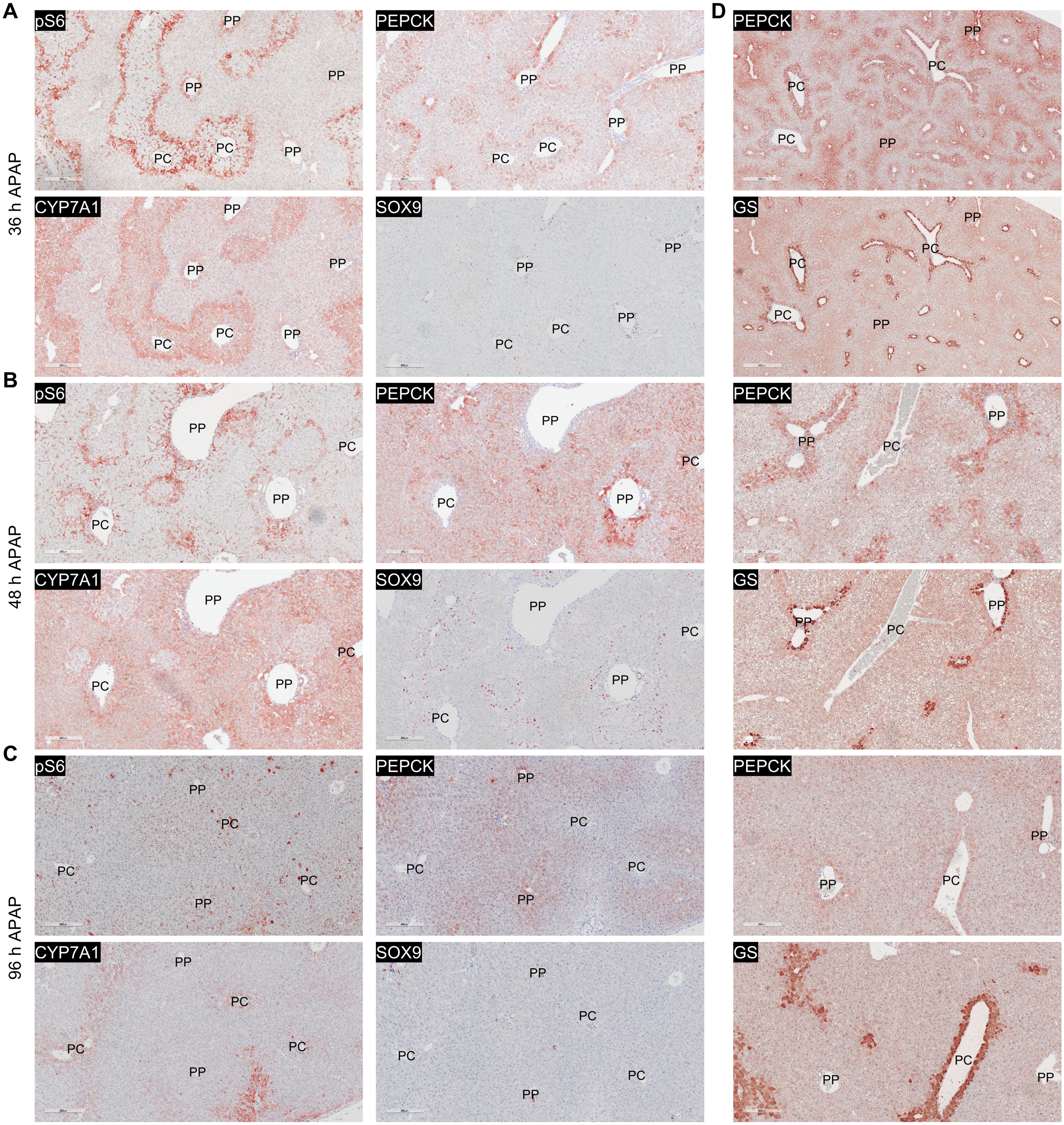

Fig. S6. pS6-positive hepatocytes emerge at the regenerating interface after acetaminophen injury. (**A** to **C**) Representative immunohistochemical images of liver sections from wild-type (WT) mice at different time points following acetaminophen (APAP) injection stained for pS6, PEPCK, CYP7A1 and SOX9. Scale bars, 200 µm. (**D**) Serial sections of the same mice were stained for PEPCK and GS. Scale bars, 500 µm. PP, periportal; PC, pericentral.

Fig. S7. Lamtor2 and Raptor deletion alter bile acid enzymes and circulating lipid profiles.

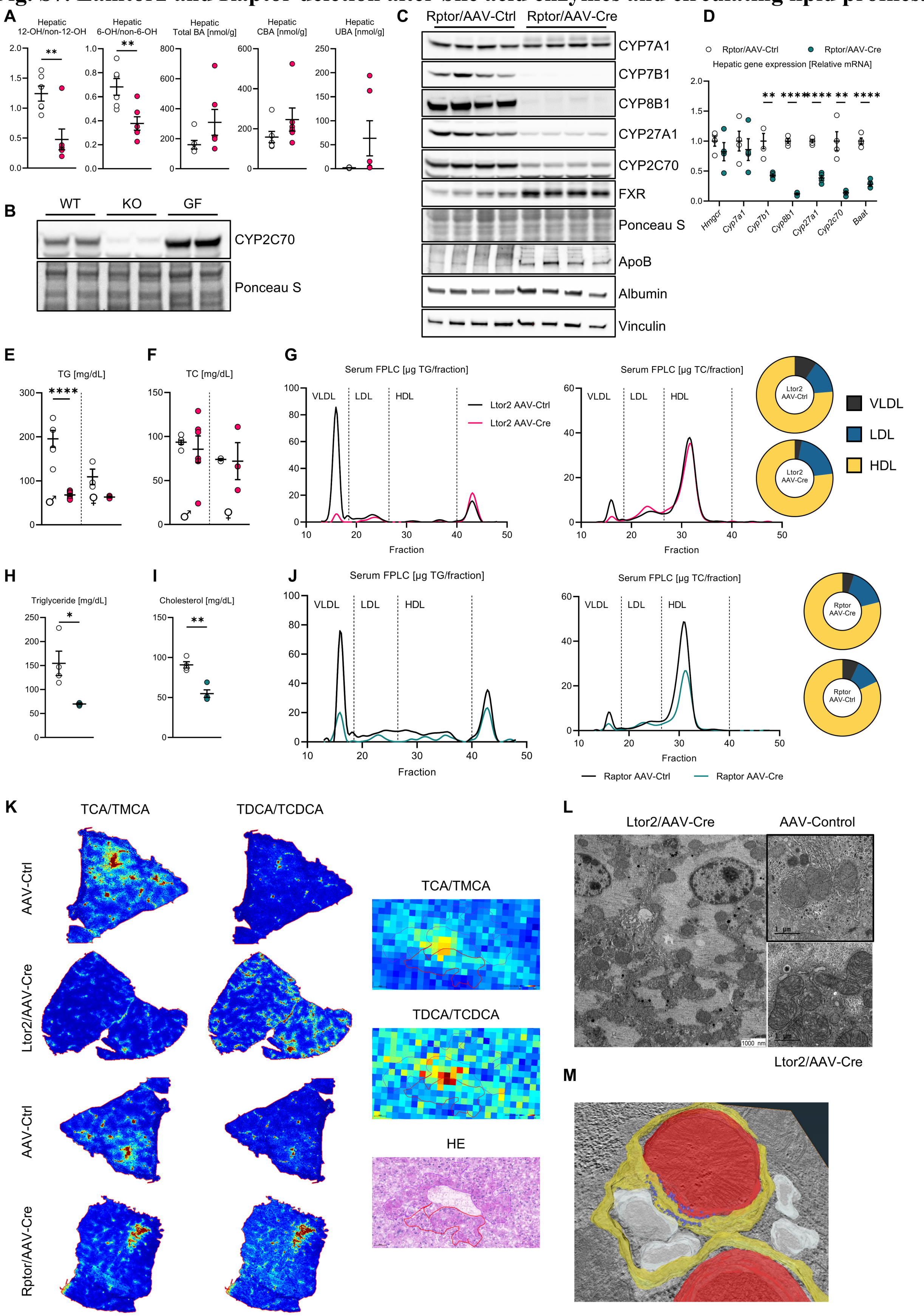

Fig. S7. Lamtor2 and Raptor deletion alter bile acid enzymes and circulating lipid profiles. (**A**) Ratios of 12-OH/non-12-OH and 6-OH/non-6-OH BAs, and concentrations of total, conjugated (CBA) and unconjugated BAs (UBA) measured in livers of male Ltor2/AAV-Ctrl compared with Ltor2/AAV-Cre four weeks after virus injection (n = 5-8). (**B**) Western blot analysis of protein expression of CYP2C70 from liver extracts of wild-type (WT), Cyp2c70 knockout (KO), and germ-free (GF) mice to validate the specificity of the antibody. (**C**) Western blot analysis of protein expression of FXR and BA biosynthetic enzymes from liver extracts of male Rptor/AAV-Ctrl compared with Rptor /AAV-Cre four weeks after virus injection (n = 4). (**D**) mRNA expression of hepatic genes involved in BA and cholesterol synthesis from livers of male Rptor/AAV-Ctrl and Rptor /AAV-Cre mice four weeks after virus injection (n = 4). (**E** to **G**) Male and female Ltor2/AAV-Cre and control mice were fed normal chow and euthanized *ad libitum* 4 weeks after virus injection to assess serum lipid homeostasis. (n = 3-6). (E) Serum analysis of triglyceride (TAG) and (F) total cholesterol (TC) concentrations. (G) Profiles of triglyceride-rich very-low-density lipoprotein (VLDL) and cholesterol-rich low-density lipoprotein (LDL) and high-density lipoprotein (HDL) by measuring triglycerides and cholesterol, respectively, in FPLC fractions (pooled plasma samples from n = 6 male mice per group). The pie chart shows the relative amount of cholesterol in VLDL, LDL, and HDL fractions. (**H** to **J**) Serum lipid parameters in male Rptor/AAV-Cre and control mice fed normal chow. (n = 4). (H) Serum analysis of triglyceride (TAG) and (I) total cholesterol (TC) concentrations. (J) Profiles of triglyceride-rich VLDL and cholesterol-rich LDL and HDL by measuring triglycerides and cholesterol, respectively, in FPLC fractions (pooled plasma samples from n = 4 male mice per group). The pie chart shows the relative amount of cholesterol in VLDL, LDL, and HDL fractions. (**K**) MALDI-MSI-based visualization and spatial distribution of trihydroxylated (TCA/TMCA) and dihydroxylated BAs (TDCA/TCDCA) in liver tissue of Ltor2/AAV-Cre, Rptor/AAV-Cre and respective controls. Zooming in on a portal field on the right shows the limits of single cell resolution with this method. (**L**) Electron micrographs of Ltor2/AAV-Ctrl and Ltor2/AAV-Cre hepatocytes. (**M**) Three-dimensional organelle reconstruction analysis demonstrating a curved, coil-like type of rough endoplasmic reticulum (yellow) associated with lysosomes (blue) and mitochondria (red). TCA, taurocholic acid; TMCA, tauro-muricholic acid; TDCA, taurodeoxycholic acid; TCDCA, taurochenodeoxycholic acid. *P < 0.05, **P < 0.01, ***P < 0.001, ****P < 0.0001. Statistical significance was calculated using a two-tailed unpaired Student's t test. Data are depicted as mean ± SEM.

Fig. S8. mTORC1 inhibition attenuates FXR target-gene activation in hepatic and intestinal cell lines.

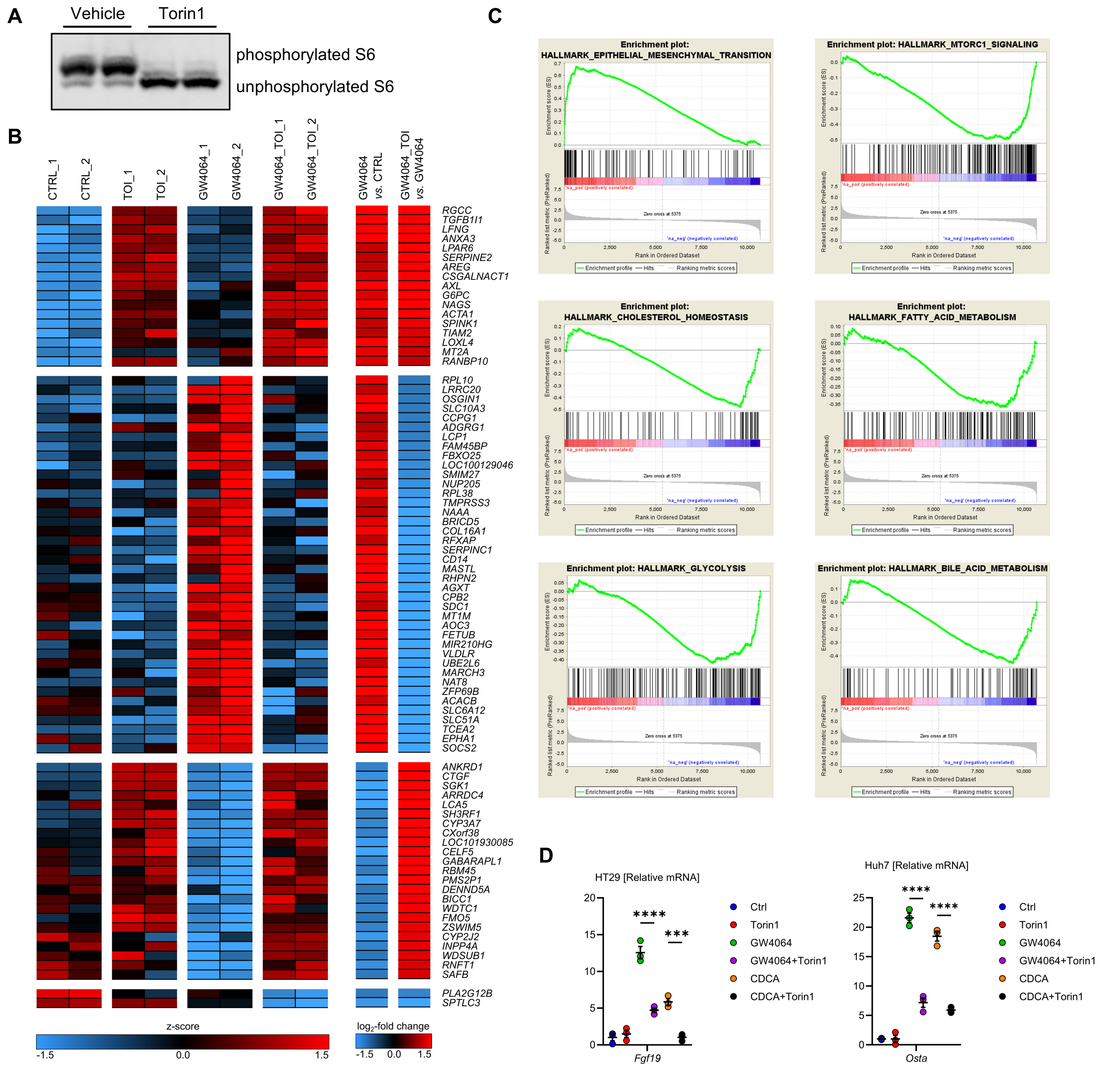

Fig. S8. mTORC1 inhibition attenuates FXR target-gene activation in hepatic and intestinal cell lines. (**A**) Phos-tag gel separation of total S6 isolated from HepG2 cells treated with either Torin1 (300 nM) or vehicle for 18 hours. (**B**) The heatmap illustrates the gene expression levels of > 2-fold up-regulated genes in GW4064 (5 μM) treated HepG2 cells versus DMSO controls, and the impact of mTORC1 inhibition by Torin1 (To1, 300 nM) on gene expression. (**C**) Gene set enrichment analysis of significantly enriched gene sets. The enrichment score (ES) reflects the degree to which a gene set was depressed with Torin1 treatment compared to DMSO. Each solid bar represents one gene within a gene set. (**D**) Induction of the FXR target genes Fgf19 in HT29 cells and Osta in Huh7 cells treated with CDCA (100 μM) and GW4064 (5 μM) in the presence or absence of the mTORC1 inhibitor Torin1 (300 nM). *$P < 0.05$, **$P < 0.01$, ***$P < 0.001$, ****$P < 0.0001$. Statistical significance was calculated using a one-way ANOVA followed by Holm-Šídák's multiple comparisons post hoc testing. Data are depicted as mean ± SEM.

Fig. S9. TFEB/TFE3 activity modifies bile acid profiles in complementary mTORC1 pathway models.

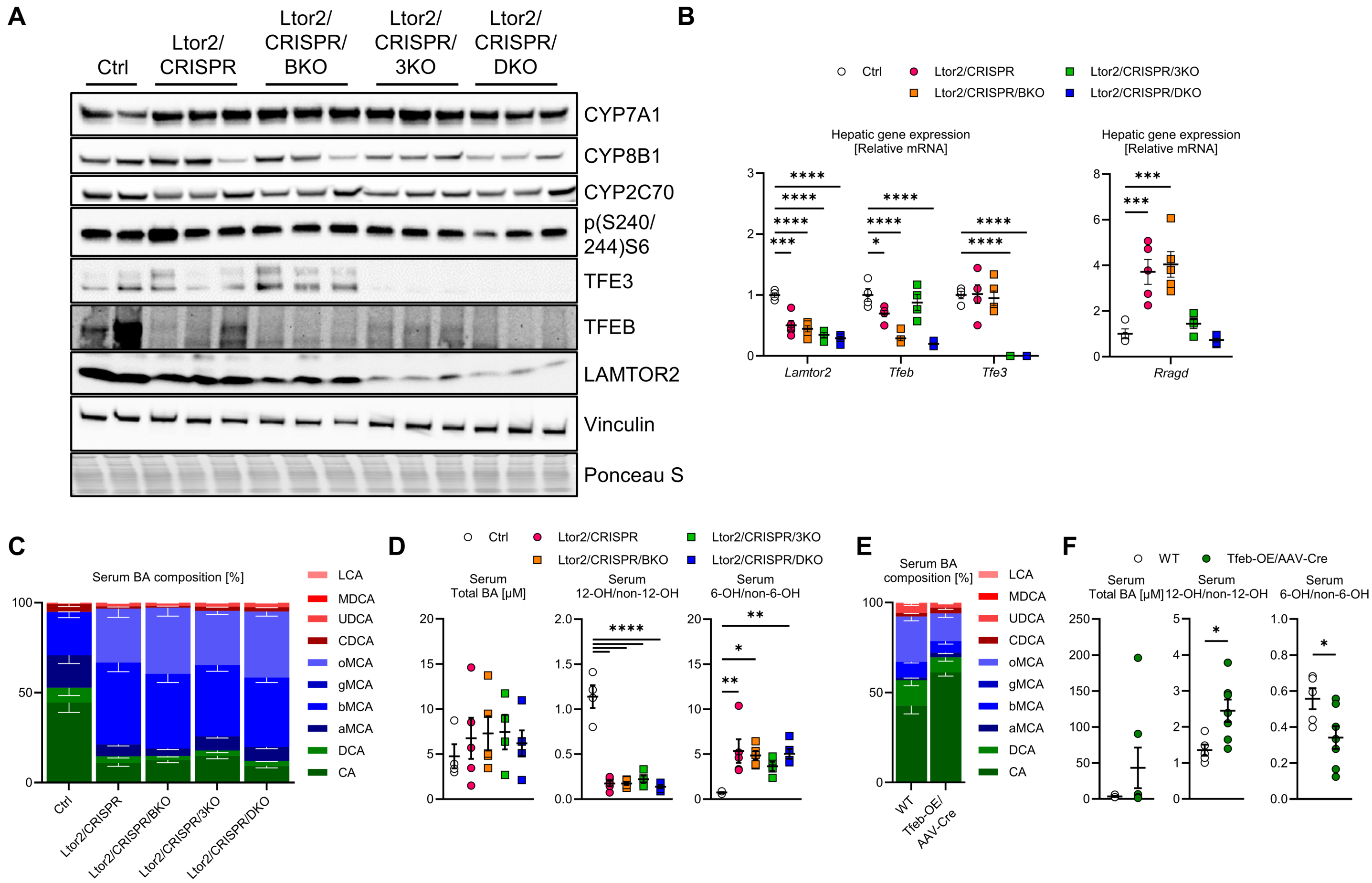

Fig. S9. TFEB/TFE3 activity modifies bile acid profiles in complementary mTORC1 pathway models. (**A**) Western blot analysis of protein expression of BA biosynthetic enzymes, phosphorylated S6 and LAMTOR2 and (**B**) confirmation of gene knockout by qPCR of Lamtor2, Tfeb and Tfe3 and Rragd from bulk liver extracts from LAMTOR2-deficient mice using CRISPR-mediated knockout together with additional knockout of TFEB (BKO) or TFE3 (3KO) or both (DKO), where indicated. Mice were euthanized five weeks after virus injection (*n* = 4-5). (**C**) Percent composition of serum BAs and (**D**) ratios of 12-OH/non-12-OH and 6-OH/non-6-OH, and total BAs in LAMTOR2-deficient mice using CRISPR-mediated knockout together with additional knockout of TFEB (BKO) or TFE3 (3KO) or both (DKO), where indicated. Mice were euthanized five weeks after virus injection (*n* = 4-5). (**E**) Percent composition of serum BAs and (**F**) ratios of 12-OH/non-12-OH and 6-OH/non-6-OH, and total BAs in TFEB-overexpressing mice, where the transgene is turned on after virus injection using AAV8-TBG-Cre (*n* = 5-7). CA, cholic acid; aMCA, alpha-muricholic acid; bMCA, beta-muricholic acid; gMCA, gamma-muricholic acid; oMCA, omega-muricholic acid; DCA, deoxycholic acid; CDCA, chenodeoxycholic acid; UDCA, ursodeoxycholic acid; MDCA, murideoxycholic acid; LCA, lithocholic acid. *$P < 0.05$, **$P < 0.01$, ***$P < 0.001$, ****$P < 0.0001$. Statistical significance was calculated using a two-tailed unpaired Student's *t* test (F) or a one-way ANOVA followed by Holm-Šídák's multiple comparisons post hoc testing (D) or a two-way ANOVA followed by Holm-Šídák's multiple comparisons post hoc testing (B). Data are depicted as mean ± SEM.

Fig. S10. Rptor-LKO and Tsc1-LKO mice show divergent systemic and hepatic bile acid profiles.

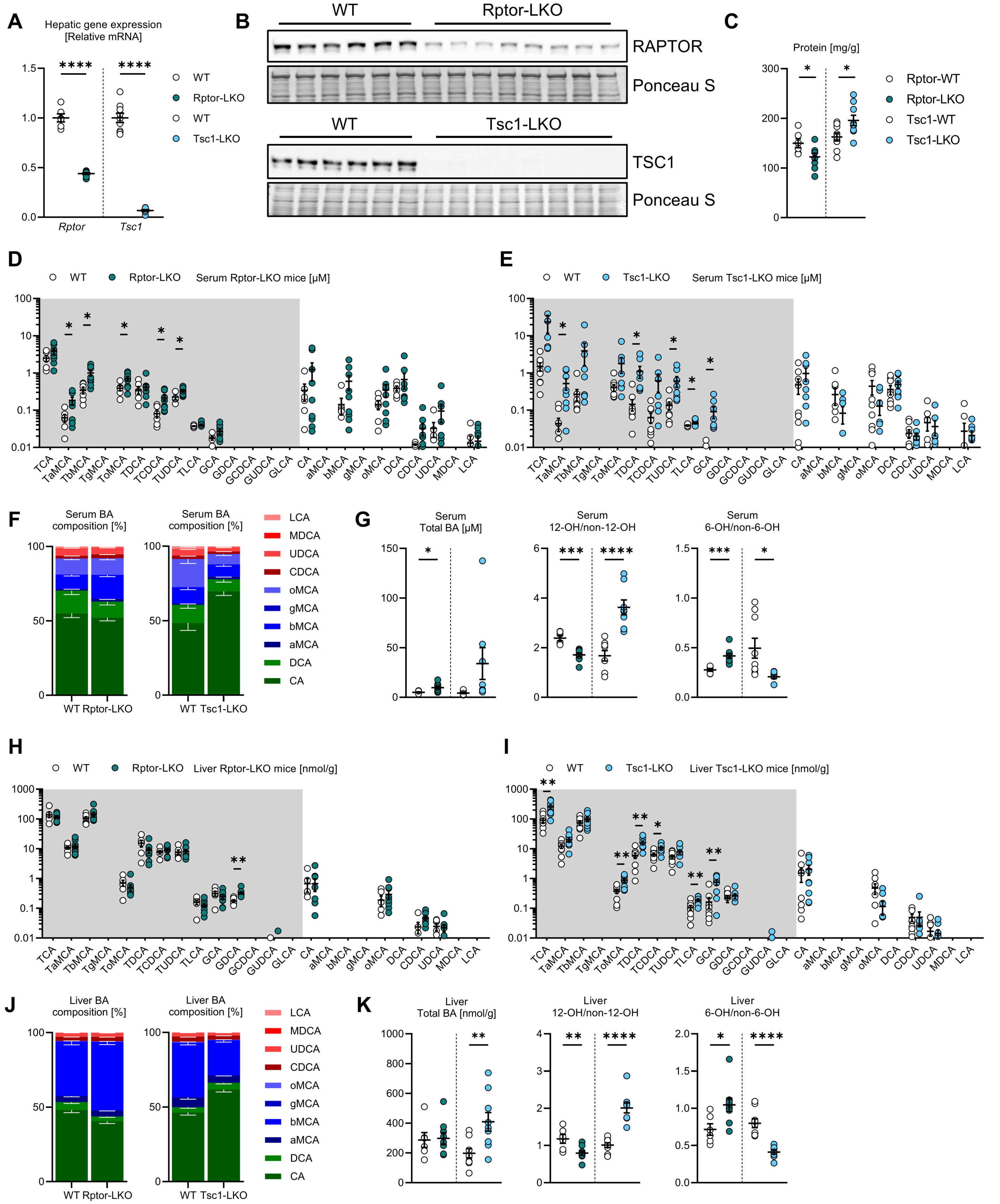

Fig. S10. Rptor-LKO and Tsc1-LKO mice show divergent systemic and hepatic bile acid profiles. (**A** and **B**) Confirmation of gene knockout by (A) qPCR of Rptor and Tsc1 and (B) immunoblotting of RAPTOR and TSC1 from bulk liver extracts obtained from Rptor-LKO and Tsc1-LKO mice, respectively. (**C**) Hepatic protein content was quantified in Rptor-LKO and Tsc1-LKO mice. (**D** and **E**) Absolute levels of bile acids, (**F**) percent composition of serum BAs and (**G**) ratios of 12-OH/non-12-OH and 6-OH/non-6-OH, and total BAs in Rptor-LKO and Tsc1-LKO mice compared to wild-type littermates (n = 6-9). (**H** and **I**) Absolute levels of bile acids, (**J**) percent composition of hepatic BAs and (**K**) ratios of 12-OH/non-12-OH and 6-OH/non-6-OH in Rptor-LKO and Tsc1-LKO mice compared to wild-type littermates (n = 6-9). CA, cholic acid; aMCA, alpha-muricholic acid; bMCA, beta-muricholic acid; gMCA, gamma-muricholic acid; oMCA, omega-muricholic acid; DCA, deoxycholic acid; CDCA, chenodeoxycholic acid; UDCA, ursodeoxycholic acid; MDCA, murideoxycholic acid; LCA, lithocholic acid. *$P < 0.05$, **$P < 0.01$, ***$P < 0.001$, ****$P < 0.0001$. Statistical significance was calculated using a two-tailed unpaired Student's t test. Data are depicted as mean ± SEM.

Fig. S11. CYP8B1 knockdown and protein restriction modify bile acid metabolism in Tsc1-LKO mice.

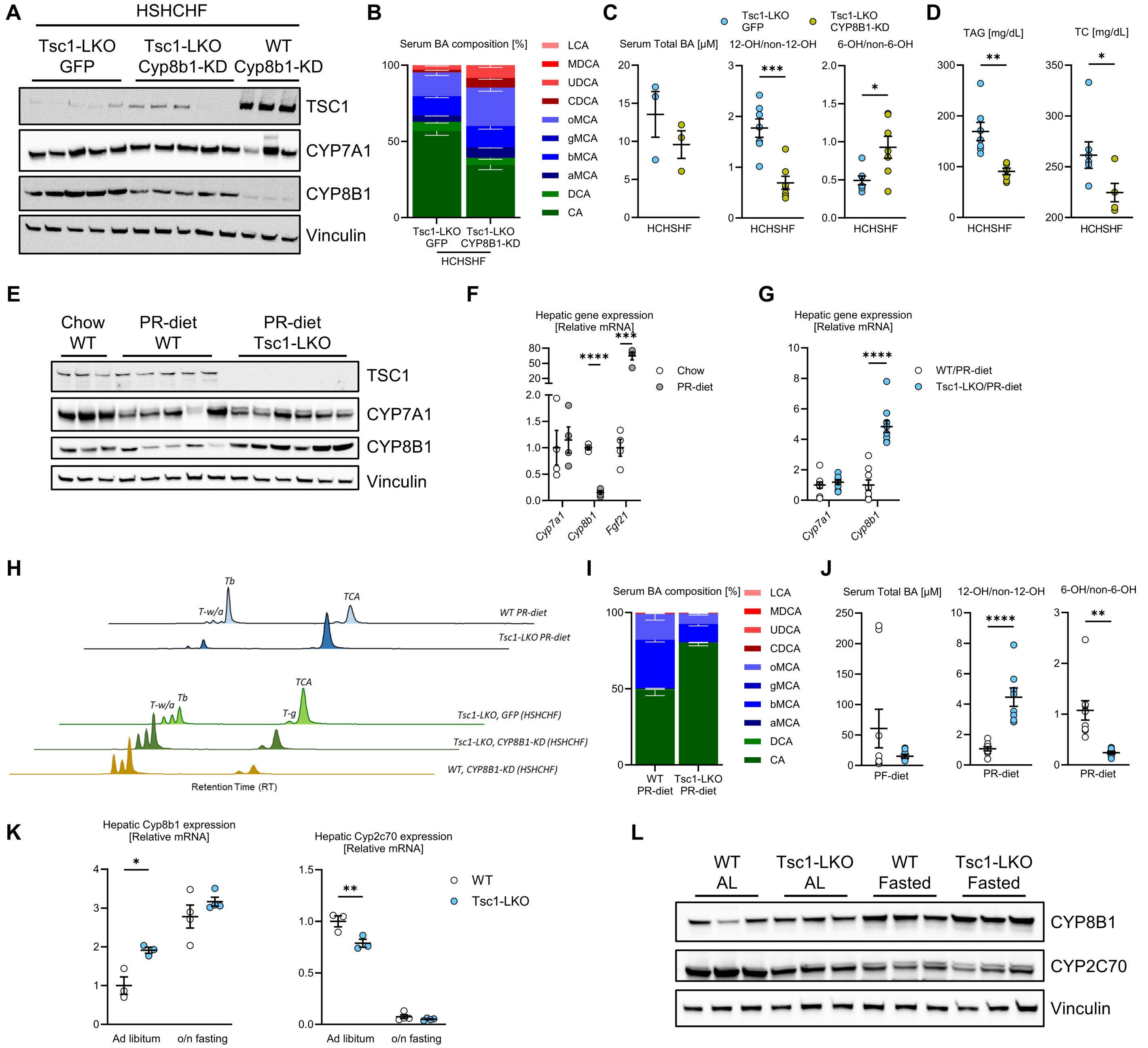

Fig. S11. CYP8B1 knockdown and protein restriction modify bile acid metabolism in Tsc1-LKO mice. (**A** to **D**) CRISPR-mediated knockdown of CYP8B1 in Tsc1-LKO and control animals on a high-sucrose/high-cholesterol/high-fat diet (HSHCHF diet). (A) Western blot analysis of protein expression of TSC2 and BA biosynthetic enzymes from bulk liver extracts. (B) Percent composition of serum BAs and (C) ratios of 12-OH/non-12-OH and 6-OH/non-6-OH, and total serum BAs. (D) Serum analysis of triglyceride (TAG) and total cholesterol (TC) concentrations. (**E**) Western blot analysis of protein expression of TSC1 and BA biosynthetic enzymes and (**F** and **G**) mRNA expression of *Cyp7a1*, *Cyp8b1* and *Fgf21* in livers from Tsc1-LKO and control mice fed normal chow or a protein-restricted diet (PR diet). (**H**) Representative total ion current chromatograms from BA measurements of the indicated genotypes and treatments. (**I**) Percent composition of serum BAs and (**J**) ratios of 12-OH/non-12-OH and 6-OH/non-6-OH, and total BAs of Tsc1-LKO and control mice on a PR diet. (**K**) mRNA expression of *Cyp8b1* and *Cyp2c70* in livers from WT and Tsc1-LKO mice either sacrificed after overnight fasting or *ad libitum* (n = 3-4). (**L**) Western blot analysis of protein expression of CYP8B1 and CYP2C70 in livers from WT and Tsc1-LKO mice either sacrificed after overnight fasting or *ad libitum*. CA, cholic acid; aMCA, alpha-muricholic acid; bMCA, beta-muricholic acid; gMCA, gamma-muricholic acid; oMCA, omega-muricholic acid; DCA, deoxycholic acid; CDCA, chenodeoxycholic acid; UDCA, ursodeoxycholic acid; MDCA, murideoxycholic acid; LCA, lithocholic acid. *$P < 0.05$, **$P < 0.01$, ***$P < 0.001$, ****$P < 0.0001$. Statistical significance was calculated using a two-tailed unpaired Student's t test (C-D,F-G,J) or a one-way ANOVA followed by Holm-Šídák's multiple comparisons post hoc testing (K). Data are depicted as mean ± SEM.

Fig. S12. Human MASLD samples link mTORC1-related signaling, bile acid composition, and protein restriction.

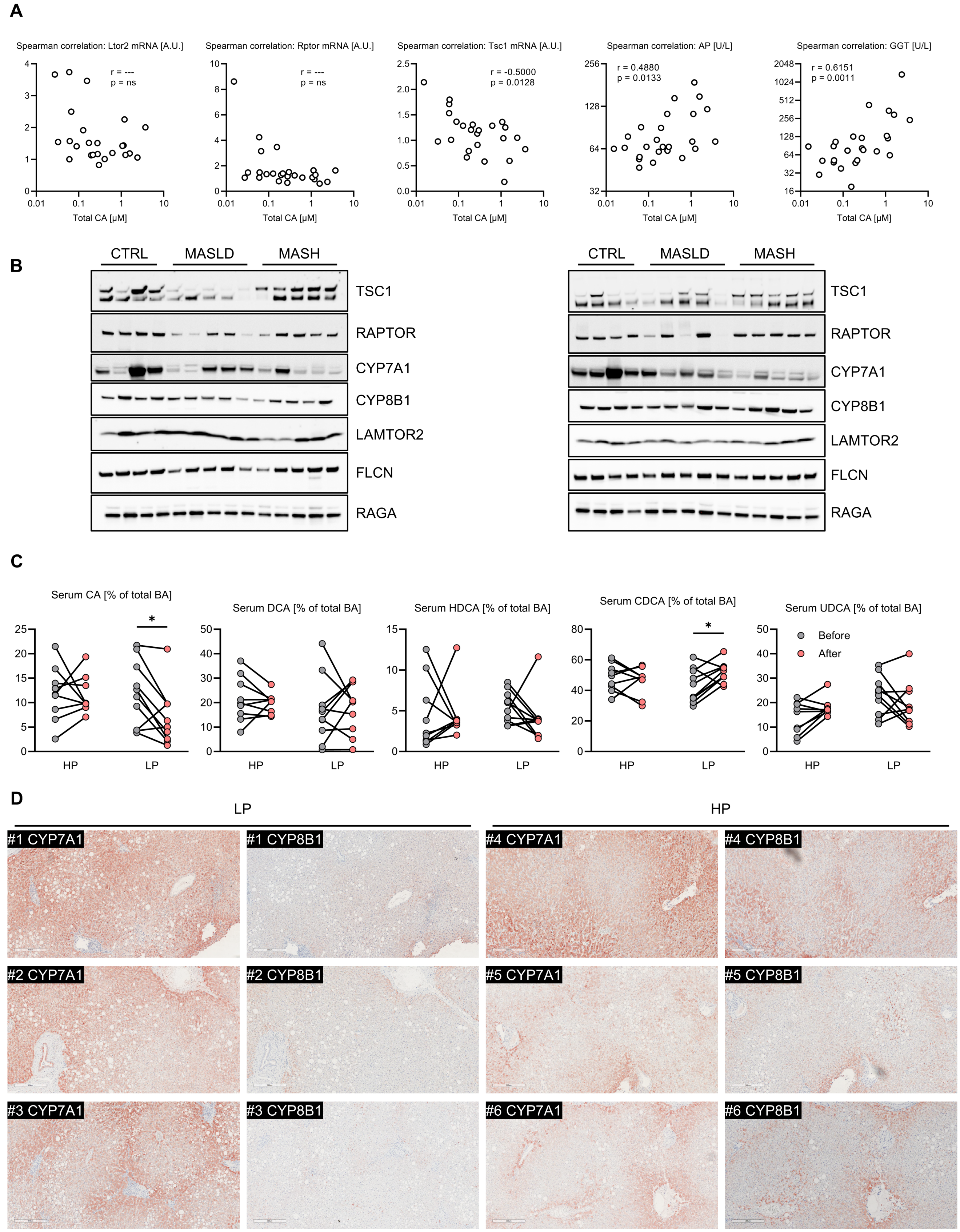

Fig. S12. Human MASLD samples link mTORC1-related signaling, bile acid composition, and protein restriction. (**A**) Spearman correlation analysis of total cholic acid (CA) serum levels in a cohort of MASLD and MASH patients with matched serum and liver samples with hepatic mRNAs and markers of liver injury. $n = 25$ patients. (**B**) Western blot analysis of protein expression of TSC1, RAPTOR, CYP7A1, CYP8B1, LAMTOR2, FLCN and RAGA in the livers of patients with MASLD and MASH compared with healthy controls (n = 8-10). (**C**) Serum BA composition in patients with MASLD on low-protein (LP) versus high-protein (HP) diets for 3 weeks. (**D**) Immunohistochemical analysis of CYP7A1 and CYP8B1 from liver sections of patients with MASLD on low protein (LP) versus high protein (HP) diets for 3 weeks. Representative images from 6 different individuals are shown. Scale bars, 300 µm. $^{*}P < 0.05$, $^{**}P < 0.01$, $^{***}P < 0.001$, $^{****}P < 0.0001$. Statistical significance was calculated using a two-tailed paired Student's t test.

Table S1. qRT-PCR primers

Gene	Forward (5' -3')	Reverse (5' -3')
	**Human**	
36b4	GCT TCA TTG TGG GAG CAG ACA	CAT GGT GTT CTT GCC CAT CAG
Fgf19	GCT CTC CAG CTG CTT CCT GC	GTG CTT CTC GGA TCG GTA CAC ATT G
Hprt	TAT GGC GAC CCG CAG CCC T	CAT CTC GAG CAA GAC GTT CAG
Lamtor2	GAC AAT CTC AAA TTC ATC CTC ATG G	CTC CAG GTA CTG CAC CAA AG
Ostα	CTA CAC CTG GGT GAG CAG AA	AGA GGA ATA GGG AGG CGA AC
Raptor	TCG TCA AGT CCT TCA AGC AG	GGG TGA TTT GGG TTG ATT GC
Tsc1	CAA CAA GCA AAT GTC GGG GAG	CAT AGG GCC ACG GTC AGA A
	**Mouse**	
36b4	GCT TCA TTG TGG GAG CAG ACA	CAT GGT GTT CTT GCC CAT CAG
Acc1	CAG ATC CAG GCC ATG TTG AG	AGA TGT GCT GGG TCA TGT GG
Acly	ACC CTT TCA CTG GGG ATC ACA	GAC AGG GAT CAG GAT TTC CTT G
Acss2	GCT TCT TTC CCA TTC TTC GGT	CCC GGA CTC ATT CAG GAT TG
Alb	GCT GAG GCC ATG TGC ACC TCC	CCA TCA AGC TTC GGG GTC AGG
Arg1	CAG AAG AAT GGA AGA GTC AG	CAG ATA TGC AGG GAG TCA CC
Baat	GCT GGT GGA TTG ATG GAG TT	AGT CGA GAA GGC AGG TCA TC
Bsep	GAG TGG TGG ACA GAA GCA AA	TGA GGT AGC CAT GTC CAG AA
Chrebpα	CGA CAC TCA CCC ACC TCT TC	TTG TTC AGC CGG ATC TTG TC
Chrebpβ	TCT GCA GAT CGC GTG GAG	CTT GTC CCG GCA TAG CAA C
Cpt1a	TGA GTG GCG TCC TCT TTG G	CAG CGA GTA GCG CAT AGT CA
Cpt1b	GGC ACC TCT TCT GCC TTT AC	TTT GGG TCA AAC ATG CAG AT
Cyp27a1	GCA CAA GGA AGT GAC TGG TG	CTG TGA TGA TCC GGG AGT TTG T
Cyp2c70	CTG TGC AGT GCC TAC CCT ATT	TCC AGA GAT TTT TGA TGC CTG TT
Cyp2e1	TCA ACC TCG TCC CTT CCA AC	AGG CCT TCT CCA ACA CAC AC
Cyp7a1	CAG GGA GAT GCT CTG TGT TCA	AGG CAT ACA TCC CTT CCG TGA
Cyp7b1	TGA AAT AGG AGC ACA TCA TCT TGG	AAT ACA TTG CCC AGA ACA TAG CTG
Cyp8b1	TTA AGG CTG GCT TCC TGA GC	TCG ACG GAA CTT CCT GAA CAG
Cyr61	AGA GGC TTC CTG TCT TTG G	TCT GAA CGA TGC ATT TCT GG
Deptor	TTT GCA ACA AAG CAG TTT GG	AAC GCA TGG CTG AAG TCC TA
F4/80	TTG GCC AAG ATT CTC TTC CT	TCA CTG CCT CCA CTA GCA TC
Fasn	GCT GTA GCA CAC ATC CTA GGC A	TCG TGT TCT CGT TCC AGG ATC
Fgf15	GAG GAC CAA AAC GAA CGA AAT T	ACG TCC TTG ATG GCA ATC G
Fgf21	TCC AAA TCC TGG GTG TCA AA	CAG CAG CAG TTC TCT GAA GC
Fxr	GAG GGC TGC AAA GGT TTC TTC	ACT TCC TGC GCA TGT ACA TGT C
G6pase	CCG GAT CTA CCT TGC TGC TCA CTT T	TAG CAG GTA GAA TCC AAG CGC GAA AC
Hes1	ACG ACA CCG GAC AAA CCA A	TCC ATG ATA GGC TTT GAT GAC TTT C
Hmgcr	CCG GCA ACA ACA AGA TCT GTG	ATG TAC AGG ATG GCG ATG CA
Hnf4α	CGG GCT GGC ATG AAG AAG G	GCA GGA GCG CGT TGA TGG
Hprt	TTG CTC GAG ATG TCA TGA AGG A	AGC AGG TCA GCA AAG AAC TTA TAG
Ibabp	CCC AAC TAT CAC CAG ACT TCG	ACA TCC CCG ATG GTG GAG AT
Igfbp1	ATC TGC CAA ACT GCA ACA AG	ACC AGC AGA GTC CAG CTT CT

Krt8	TCT GGG ATG CAG AAC ATG AG	GCT CAT TCC GTA GCT GAA GC
Lamp1	CAG CAC TCT TTG AGG TGA AAA AC	CCA TTC GCA GTC TCG TAG GTG
Lamtor2/p14	TCT GGG CCG CGT ATG ATA GG	CAC GCT GTT ATG ATG CTG CTA CTT
Ostβ	GAC AAG CAT GTT CCT CCT GAG	GAT GCA GGT CTT CTG GTG TTT C
Pck1	TGG GAA TCT CCA GAA GGA GT	GGA CAC CTT CAG GTC TAC GG
Pparg1	GAT TTG AAA GAA GCG GTG AAC C	GCA TCT CTG TGT CAA CCA TGG T
Pparg2	TGC CTA TGA GCA CTT CAC AAG AAA T	CGA AGT TGG TGG GCC AGA A
Raptor	CAG AGG GAG CAG GAA CTA GA	TTT CAT TGA GGG AGG CAA GG
Rictor	TGC GAT ATT GGC CAT AGT GA	ACC CGG CTG CTC TTA CTT CT
Rraga	GAA CCT GGT GCT GAA CCT GT	GAT GGC TTC CAG ACA CGA TT
Rragc	AGA TGT CAC CCA ATG AGA CTC T	AGT CGT CCT GTG CAT CAA TGA
Rragd	CTG TTT GAC GTG GTC AGT AAG AT	GTT GAG TCC TTG TCA TAC GGG
Scd1	ATC GCC TCT GGA GCC ACA G	ACA CGT CAT TCT GGA ACG CC
Shp	AAG GGC ACG ATC CTC TTC AA	GTA CCA GGG CTC CAA GAC T
Srebp1a	GGC CGA GAT GTG CGA ACT	TTG TTG ATG AGC TGG AGC ATG T
Srebp1c	GGA GCC ATG GAT TGC ACA TT	GGC CCG GGA AGT CAC TGT
Sox9	CGA GGA AGA TAA GTT CCC CGT GTG C	CAG CAG CCT CCA GAG CTT GCC C
Spp1	ACA CTT TCA CTC CAA TCG TCC	TGC CCT TTC CGT TGT TGT CC
tdTom	GTG ACC GTG ACC CAG GAC	CGC GCA TCT TCA CCT TGT
Tfe3	AAC AGC AAC GCT CCA AAG ACC	AGA CCG AAC TCG TGG TTA GGG
Tfeb	AGG TCA CAG CCT CCA TGG TG	TCT TCT GCC GCT CCT TGG C
Uxt	CTC ACA GAG CTC AGC GAC AGC	AAA TTC TGC AGG CCT TGT AGT TCT C
